# Using Genetics to Inform Interventions Related to Sodium and Potassium in Hypertension

**DOI:** 10.1161/CIRCULATIONAHA.123.065394

**Published:** 2023-12-21

**Authors:** William R. Reay, Erin Clarke, Shaun Eslick, Carlos Riveros, Elizabeth G. Holliday, Mark A. McEvoy, Roseanne Peel, Stephen Hancock, Rodney J. Scott, John R. Attia, Clare E. Collins, Murray J. Cairns

**Affiliations:** 1 Schools of Biomedical Sciences and Pharmacy (W.R.R., R.J.S., M.J.C.), The University of Newcastle, Callaghan, NSW, Australia.; 2 Health Sciences (E.C., S.E., C.E.C.), The University of Newcastle, Callaghan, NSW, Australia.; 3 Medicine and Public Health (E.G.H., R.P., S.H., J.R.A.), The University of Newcastle, Callaghan, NSW, Australia.; 4 Precision Medicine Research Program (W.R.R., M.J.C.), New Lambton, NSW, Australia.; 5 Food and Nutrition Research Program (E.C., C.E.C.), New Lambton, NSW, Australia.; 6 Cancer Detection and Therapy Research Program (R.J.S.), New Lambton, NSW, Australia.; 7 Hunter Medical Research Institute (C.R., E.G.H., J.R.A.), New Lambton, NSW, Australia.; 8 Rural Health School, La Trobe University, Bendigo, Victoria, Australia (M.A.M.).

**Keywords:** genetics, hypertension, potassium, precision medicine, sodium

## Abstract

**BACKGROUND:**

Hypertension is a key risk factor for major adverse cardiovascular events but remains difficult to treat in many individuals. Dietary interventions are an effective approach to lower blood pressure (BP) but are not equally effective across all individuals. BP is heritable, and genetics may be a useful tool to overcome treatment response heterogeneity. We investigated whether the genetics of BP could be used to identify individuals with hypertension who may receive a particular benefit from lowering sodium intake and boosting potassium levels.

**METHODS:**

In this observational genetic study, we leveraged cross-sectional data from up to 296 475 genotyped individuals drawn from the UK Biobank cohort for whom BP and urinary electrolytes (sodium and potassium), biomarkers of sodium and potassium intake, were measured. Biologically directed genetic scores for BP were constructed specifically among pathways related to sodium and potassium biology (pharmagenic enrichment scores), as well as unannotated genome-wide scores (conventional polygenic scores). We then tested whether there was a gene-by-environment interaction between urinary electrolytes and these genetic scores on BP.

**RESULTS:**

Genetic risk and urinary electrolytes both independently correlated with BP. However, urinary sodium was associated with a larger BP increase among individuals with higher genetic risk in sodium- and potassium-related pathways than in those with comparatively lower genetic risk. For example, each SD in urinary sodium was associated with a 1.47–mm Hg increase in systolic BP for those in the top 10% of the distribution of genetic risk in sodium and potassium transport pathways versus a 0.97–mm Hg systolic BP increase in the lowest 10% (*P*=1.95×10^−3^). This interaction with urinary sodium remained when considering estimated glomerular filtration rate and indexing sodium to urinary creatinine. There was no strong evidence of an interaction between urinary sodium and a standard genome-wide polygenic score of BP.

**CONCLUSIONS:**

The data suggest that genetic risk in sodium and potassium pathways could be used in a precision medicine model to direct interventions more specifically in the management of hypertension. Intervention studies are warranted.

**Clinical Perspective**

**What Is New?**

- This is the first study to explore whether genetic risk scores could identify individuals for whom blood pressure is affected differently by sodium and potassium.
- Sodium was associated with larger hypertensive effects for individuals with higher blood pressure genetic risk in pathways related to sodium and potassium biology.

**What Are the Clinical Implications?**

- Genetic risk scores in sodium and potassium pathways may prove useful to identify individuals who respond differently to interventions targeting these electrolytes.
- Intervention studies are warranted to examine the usefulness of these genetic risk scores to inform precision prevention and management of hypertension.

Hypertension affects ≈1.3 billion adults >30 years of age worldwide.^1^ Risk for hypertension has a strong genetic component; modifiable components of risk include diet, particularly excess intake of sodium and inadequate intake of potassium-rich foods such as fruits and vegetables.^2,3^ Sodium (Na^+^) intake increases blood pressure (BP) through a number of mechanisms, such as increasing plasma renin activity and angiotensin II, as well as stimulating the sympathetic nervous system.^3^ Potassium intake lowers BP by stimulating vasodilation through endothelial cell hyperpolarization,^4,5^ along with renal mechanisms related to electrolyte homeostasis in distal segments of the nephron.^6–8^ There is a large body of evidence showing that adjusting an individual’s ratio of sodium to potassium intake can influence BP,^9–12^ with specific recommendations that the ideal ratio of sodium to potassium is 1:1 or lower.^13^ Higher sodium-to-potassium ratios are also associated with poorer diet quality that aligns more with a Western, or unhealthier, dietary pattern.^14^ However, potassium intake may not be purely antihypertensive, with evidence that excessive intake can increase BP.^7,15,16^

Despite strong evidence supporting shifting sodium-to-potassium ratios lower toward increased potassium in the prevention and treatment of hypertension, there is significant heterogeneity in response to dietary interventions related to sodium and potassium.^15,17^ This heterogeneity is likely heavily influenced by the biological complexity of the pathogenesis of hypertension. Hypertension, as a dichotomous trait, and BP, as a continuous variable, are complex traits that emerge from an interplay between genetic and environmental factors. Genome-wide association studies (GWAS) have indicated that hypertension is a polygenic trait, whereby mostly small effect size variants influence hundreds, if not thousands, of genes that play a role in its pathogenesis.^18^ These data have provided genetic support for specific pathways, such as transforming growth factor–β signaling,^18^ as well as evidence of shared genetic risk with other clinically relevant traits.^19^ Genome-wide polygenic scores (PGSs) for hypertension that aggregated genetic risk burden in an individual have shown some promise as a tool for risk stratification.^20,21^ However, a key limitation of conventional genetic risk scoring approaches is a lack of clear biological insights at the individual level because of their construction from heterogenous variants genome-wide, and thus, they lack biological specificity in most instances regarding formulating treatments or interventions. We previously sought to overcome this limitation through the development of the pharmagenic enrichment score (PES) platform.^22–26^ The PES is a PGS that is specifically constructed among pathways or networks that can be modulated by an intervention, rather than genome-wide, with the underlying hypothesis that individuals with heightened genetic risk in pathways relevant to the treatment may respond more readily. The PES is extremely flexible in the sense that it can be used for almost any outcome and treatment combination provided that sufficient genetic data are available and the intervention has appreciable biology. We previously demonstrated that this platform reveals distinct biological insights relevant to treatment, as well as potentially providing a mechanism whereby some individuals with low overall genome-wide risk still develop the disorder or manifest an extreme population deviation for continuous traits.^22,24–26^ The PES approach could also be applied to dietary interventions, as genetic risk could be constructed among specific biology known to be related to said intervention.

In this study, we sought to investigate whether sodium- and potassium-directed PES for BP may be useful to identify individuals who may receive a particular antihypertensive benefit from lowering sodium and increasing potassium compared with a genome-wide genetic scoring approach. These genetic approaches were explored in conjunction with objective urinary biomarkers of sodium and potassium intake to establish whether there is evidence for a gene-by-environment interaction.

## METHODS

Because of the sensitive nature of the data collected for this study, requests to access the data set from qualified researchers trained in human subject confidentiality protocols must be sent to the UK Biobank (UKBB) access committee (http://amsportal.ukbiobank.ac.uk). UKBB data used in this study are available to researchers after approval by the UKBB access management committee.

### Overview

We aimed to investigate the effect of genetics on the relationship between sodium and potassium intake (as indexed by urinary electrolytes) and BP. Genome-wide PGSs were generated for systolic and diastolic BP, which represent common variant influences on BP across the genome in an individual. By way of comparison, we generated pharmagenic enrichment scores (PESs) in sodium and potassium pathways that subset the total BP genetic signal captured by PGS. We then investigated the magnitude of the association between PGS and PES on measured BP. Gene-by-environment interaction modeling was subsequently performed to investigate whether sodium and potassium PES could identify individuals who may receive an outsized benefit from interventions related to modifying sodium and potassium levels.

### Study Design

This study is primarily a cross-sectional, observational study of 2 genotyped cohorts with measured BP available: the Hunter Community Study (HCS) and the UKBB.^27,28^ The HCS is a population-based cohort of middle-aged to elderly individuals (55 to 85 years of age at recruitment) residing in and around the region of Newcastle, New South Wales, on the east coast of Australia. HCS participants were recruited by random selection from the New South Wales State electoral roll, described in detail previously.^27^ Use of the HCS data was approved by the University of Newcastle human ethics research committee (reference H-820-0504a). The UKBB is a population-based longitudinal study of ≈500 000 individuals residing in the United Kingdom who were between 40 and 69 years of age at recruitment.^28^ Use of these data was approved by the UKBB access management services system (project ID 58432). The primary aim of this study was to explore the interplay between estimated sodium and potassium intake (as reflected by urinary electrolytes and dietary questionnaires) and genetics on BP, with genetic risk considered using a genome-wide approach and a PES related to sodium and potassium biology.^22–26^

### Genotyping and Imputation

The UKBB single nucleotide polymorphism (SNP) genotyping procedure and platforms have been outlined extensively previously, with DNA extracted from blood at the baseline visit of participants to a UKBB assessment center.^28^ We processed and performed quality control on the imputed genotype data of this cohort in a previous study, with an additional description in the Methods in the Supplemental Material.^24^ This pipeline resulted in retaining 336 896 unrelated White participants of British ancestry, based on kinship estimation and principal component analysis, respectively. The genotyping, imputation, and quality control of the HCS has been described extensively previously.^25,27^ This resulted in 2089 unrelated participants of predominantly European genetic ancestry with imputed genotypes available.

### Estimating the Cross-Sectional Effect of Urinary Electrolytes on BP

In the UKBB, we focused on the time point when individuals attended one of the UKBB assessment centers for the first time across the United Kingdom for the following: study nurse interview and questionnaire completion by touchscreen, a blood draw, a spot urine sample, or anthropometric measurements. We retained participants with nonmissing automated systolic BP (SBP) and diastolic BP (DBP) measures (mean of all measures in mm Hg) who also had genotyping data available after quality control. Individuals who reported taking an antihypertensive medication at the time of assessment (field IDs 6153 and 6177), and thus had BP measurement, were also identified for downstream adjustment. This cohort was then further filtered to only retain individuals with nonmissing urinary biomarker measurements (sodium, potassium, and creatinine), with a spot urine measurement provided at the same time point. Urinary electrolytes were analyzed using the potentiometric method, and creatinine was measured enzymatically, all using a single Beckman Coulter AU5400 clinical chemistry analyzer with manufacturer reagents. The urinary sodium (UNa^+^) and urinary potassium (UK^+^) concentrations are reported in units of millimoles per liter, whereas creatinine is reported in units of micromoles per liter. A single uniform volume of urine was held and analyzed by the UKBB for all participants (5100 μL). Although these are spot measurements of UNa^+^ and UK^+^, it has been shown that spot samples perform relatively well compared with 24-hour urine collection, particularly in large samples.^29,30^ However, spot samples are affected by various factors, including fasting time before urine collection, which is an important limitation of using these measurements. Individuals with missing baseline body mass index (BMI) were excluded. We excluded a small number of individuals with urinary electrolyte measurements outside the detectable range of the assay, as flagged by the UKBB. This resulted in a full study cohort of 296 475 participants. As a sensitivity analysis, we estimated variance explained (adjusted coefficient of variation) by each of the following plausible confounders in a separate linear model on raw and natural log-transformed SBP, DBP, UNa^+^, and UK^+^, respectively: age, sex, assessment center attended, seasonality of measurement (month attended assessment center), 20 SNP-derived principal components, BP medication as a binary indicator, and BMI (Figure S1).

Cross-sectional correlation between urinary electrolytes and BP was estimated using multiple linear regression models. The primary models were adjusted for age, sex, assessment center attended, seasonality (month attended assessment center), 20 SNP-derived principal components, and antihypertensive medication use. We also tested the same models in individuals not using antihypertensive medication (n=235 436). We then constructed follow-up models in both the full and unmedicated cohort that also adjusted for BMI, as well as both BMI and urinary creatinine.

### Genome-Wide Polygenic Risk Scoring

To aggregate the burden of BP-associated genetic association at an individual level, we constructed genome-wide PGSs for SBP and DBP, as described in the Methods in the Supplemental Material.^31,32^ A GWAS of these 2 phenotypes was obtained that was independent of the HCS and UKBB to weight the scores from the Genetic Epidemiology Research on Adult Health and Aging cohort.^33^ The GWAS was performed on a subset of this cohort of majority European ancestry with relevant BP data available (up to 99 785 participants).

We then profiled SBP and PGSs at different *P* value thresholds for inclusion of independent genetic variants in the HCS score. This was performed for the purpose of tuning (ie, selecting the threshold that explained the most phenotypic variance in BP for subsequent testing in the UKBB). An independent cohort from the UKBB was used for tuning the scores to avoid overfitting of genetic effects.^34,35^ The final study cohort in the HCS comprised 1833 unrelated individuals of genetically inferred European ancestry, as described previously, filtered to retain those with nonmissing BP measurements and data available related to antihypertensive medication use. We further split the cohort into a subset of participants who did not report taking an antihypertensive medication at baseline (n=852) and those who did (n=981). In each subcohort, we separately estimated the phenotypic variance explained by each PGS on the respective outcome (SBP or DBP) as indexed by the coefficient of determination (*R*^2^). Δ*R*^2^ was calculated from comparing 2 multiple linear regression models (*R*^2^_Full_−*R*^2^_Null_). The null model included the covariates alone (age, sex, and 5 SNP-derived principal components); the full model added the PGS coefficient. We averaged the Δ*R*^2^ estimated in the medicated and unmedicated cohorts to select the optimal PGS configuration for SBP and DBP to take forward to profile in the UKBB. Plink2 v2.00a3.1LM 64-bit was used to generate the PGS.

### Constructing PESs for BP Annotated to Sodium and Potassium Biology

PESs for SBP and DBP were then constructed specifically in biology and pathways related to sodium and potassium biology. This involved identifying gene lists related to sodium and potassium biology. We achieved this by searching the MSigDB (molecular signatures database) for ontological pathways related to these factors. Genes from relevant pathways were collated in the following categories (Tables S1 and S2): nutrient absorption, transport (sodium, potassium, and both sodium and potassium), and renal excretion (sodium, potassium, and both sodium and potassium), totaling 7 sets of genes used to construct and tune PESs for SBP and DBP. The SBP and DBP summary statistics were filtered for variants overlapping the genes in each pathway using a custom script for this purpose that was previously developed by our group (https://github.com/Williamreay/Pharmagenic_enrichment_score/blob/master/Filter_pathway_variants.py). Profiling and tuning in the HCS of these scores was analogous to the genome-wide PGS, as outlined in the Methods in the Supplemental Material. We selected the best performing scores from each of absorption, transport, and renal excretion categories to take forward to the UKBB.

### Baseline Effects of PGSs and PESs in the UKBB

The tuned PGS and absorption, transport, and renal excretion PES were profiled in the UKBB cohort as described previously using plink2. We first tested the main effects of each PGS and PES on their respective BP measurements using the same multiple linear regression model as the urinary electrolytes and BP analyses, but with a genetic term instead of the urinary metabolite and the addition of 20 SNP-derived principal components to control for residual ancestral effects among this White British portion of the UKBB. Analyses were performed in both the full and unmedicated cohorts, and scores were standardized to have a mean of 0 and unit variance for the purposes of effect size interpretation. To guard against nonspecific inflation of the BP genetic signal driving associations between PES and BP, we repeated those PES models additionally adjusted for genome-wide PGS.^22,24–26^

### Investigation of Nonadditive Effects (Gene-by-Environment Interaction)

We then hypothesized that the joint effect of genetic propensity for BP, either indexed as a PGS or a biologically annotated PES, with the urinary electrolytes may depart significantly from additivity and, therefore, constitute a polygenic (gene)–by-environment interaction (G×E). In other words, we tested whether genetics modifies the relationship of urinary electrolytes with BP. These analyses were primarily examined in the unmedicated cohort, as this subset exhibited more variability in BP, and thus, we are likely better powered to detect nonadditive effects. Moreover, antihypertensive medications (particularly diuretics, which affect renal excretion) are known to influence urinary electrolytes.^36^ Full details of the modeling procedure are outlined in the Methods in the Supplemental Material. We first screened the best-performing PGS and PES for SBP and DBP in a model that added an interaction term between the urinary electrolyte and the genetic score ($\beta_{G\times E}$). PES or PGS with a nominally significant (*P*<0.05) G×E term with either UNa^+^ or UK^+^ were then carried forward for additional sensitivity analyses. As shown previously, spurious G×E effects can be detected when interaction terms between the environmental exposure of interest (UNa^+^ or UK^+^) are not included with all covariates, as well as interaction terms between the genetic term and all covariates.^37^ To address this, we constructed 2 additional models for G×E pairs with some evidence for nonadditivity that controlled for gene-by-covariate (G×C) effects and both G×C and covariate-by-environment (C×E) effects.

For the most significant G×E effect detected in both models, we performed additional sensitivity analyses. First, we estimated the effect of the UNa^+^ on BP at each decile of either the PES or PGS to screen for evidence that the sodium effect size is significantly different between these groups. The slopes of the electrolyte effect among participants in the top decile (${\hat{\beta }}_{10}$) versus each other decile (${\hat{\beta }}_{k}$ were then sequentially tested for statistically significant differences using a *Z* test as outlined elsewhere.^38^ To better capture the effects of urinary electrolytes across the distribution of the genetic scores, we then split the cohort into percentiles of PES or PGS. Estimating the association of the urinary electrolyte at the resolution of percentiles allowed us to fit smoothed linear and nonlinear curves of the relationship between increasing genetic risk and the association of the urinary electrolyte with BP. We also tested the effect of adjusting for urinary creatinine, as well as estimating interactions in the full cohort that covary for medication status. The statistical significance of G×E effects are also particularly prone to inflation because of heteroskedasticity,^39^ and as such, we reestimated SEs as heteroskedasticity consistent (HC) SEs, specifically leveraging the HC0 (White’s estimator) and HC3 methods using the sandwich R package v3.0-1.^40,41^ We then considered whether the *G* term of interest (PGS or PES) was associated with differences in BP variance, rather than just mean effects. Genetic correlates with the variance of quantitative traits have previously been shown to be enriched for factors that display detectable G×E effects.^42,43^ We tested this by splitting the relevant score into quantiles followed by testing for significant differences in variance between these quantiles using Levene’s test, as outlined in the Methods in the Supplemental Material. We also explored the influence of renal function and urinary dilution on the most significant interaction finding. As outlined in the Methods in the Supplemental Material, this was achieved through consideration of estimated glomerular filtration rate (eGFR) using age, sex, and serum cystatin C,^44–46^ as well as benchmarking the urinary electrolytes to urinary creatinine (ie, a urinary electrolyte-to-urinary creatinine ratio).

### Investigating the Influence of Diet Quality

We then investigated an index of overall quality of dietary consumption based on self-report versus the very specific intake patterns of sodium and potassium explored in the previous sections using the urinary biomarkers. Dietary intake data were collected using the Oxford WebQ, a validated 24-hour dietary questionnaire that reports the frequency of consumption of 206 foods and 32 beverages during the previous 24 hours.^47–49^ Dietary intake data were collected between April 2009 and June 2012 and repeated 4 times, totaling 5 collections. For the first collection, participants completed the Oxford WebQ at the assessment center using a touch screen, which is the time point of interest in this study. Participants had 3 days to complete the questionnaire for the first 2 repeated cycles and 14 days for the last 2 repeated cycles. Nutrient calculations involved multiplying the frequency of consumption of reported foods and beverages by a standard portion size and the nutrient composition of each food and beverage.

Diet quality was calculated using recommended food score (RFS) indices.^50^ This scoring approach assigns points for intake of 5 food groups: fruits, vegetables, whole grains, low-fat dairy, and lean meats or alternatives. Scoring was based on the methods outlined by Livingstone et al.^51^ Food groups were scored at 1 point for minimum consumption, defined as 15 grams of intake for foods and 30 grams for beverages. Zero points were given if the food was consumed under this amount or if NA was reported. The scores ranged from 0 to 21. Higher scores are indicative of a greater consumption of the 5 food groups, and therefore, higher diet quality. We then identified individuals in our primary study cohort with an RFS at baseline who did not self-report that their diet yesterday was not their usual diet. The cross-sectional correlation between RFS and BP/urinary electrolytes was tested using the same multiple linear regression model for the cross-sectional BP/electrolyte model described previously. The G×E interaction modeling with standardized RFS (mean 0, unit variance) was performed as with the urinary electrolytes, testing the 3 genetic scores that demonstrated at least nominal evidence of an interaction with UNa^+^ or UK^+^.

### Statistical Analysis

The statistical analyses performed in this study are described in detail throughout the Methods. In summary, the cross-sectional relationship between urinary electrolytes and BP was tested using multiple linear regression, with the differing covariates described in detail in Estimating the Cross-Sectional Effect of Urinary Electrolytes on BP. Genetic risk scores (PGS and PES) were also tuned using multiple linear regression, whereby the largest coefficient of determination (*R*^2^) was used to select the optimal score configuration. G×E interactions were tested through adding nonadditive interaction terms to the multiple linear regression models. Sensitivity analyses for the G×E results were undertaken through estimating heteroskedasticity-consistent SEs and formally comparing the BP effects of the electrolyte at different quantiles of genetic risk using a *Z* test. Variance in BP between genetic risk quantiles was assessed using Levene’s test. The cross-sectional relationship between diet quality and BP was also tested using multiple linear regression.

### Data Sharing and Availability

The UKBB data set is available upon approval by the UKBB data access committee, with detailed information on how to apply for access provided elsewhere (https://www.ukbiobank.ac.uk/enable-your-research/apply-for-access). GTEx data are available to all researchers upon direct application (https://www.gtexportal.org/home/datasets). Code used in this study is available on GitHub (https://github.com/Williamreay/Na_K_BP_PES). All remaining data are available as outlined throughout the text.

## RESULTS

### Cross-Sectional Relationship Between Spot Urinary Electrolytes and BP in the UKBB

In line with expectations, we demonstrated consistent evidence that urinary electrolytes were significantly correlated with SBP and DBP in the UKBB (Table S3). In the full cohort, each mmol/L in spot UNa^+^ was associated with a statistically significant increase of 0.025 mm Hg (95% CI, 0.024, 0.026) and 0.015 mm Hg (95% CI, 0.014, 0.016) in SBP and DBP, respectively. Spot UK^+^ exhibited the converse, with each mmol/L associated with a 0.04 mm Hg (95% CI, 0.038, 0.042) and 0.009 mm Hg (95% CI, 0.008, 0.01) decrease in SBP and DBP, respectively. We observed that these observational relationships were consistent considering only participants who did not self-report antihypertensive use at baseline, as well as after BMI and urinary creatinine were added as covariates or the electrolytes were natural log-transformed (Table S3). For example, among individuals not taking antihypertensives, each mmol/L in UNa^+^ exhibited a very similar effect size on SBP of 0.028 mm Hg (95% CI, 0.026, 0.029). In terms of the ratio of UNa^+^ to UK^+^, we saw a monotonic elevation in the observed positive relationship with BP as the ratio shifted above 1 to be more sodium-dominant (Figure S2). These data provide a baseline observational estimate of the correlation between these urinary electrolytes and BP for subsequent genetic stratification; however, there are many other plausible confounders of the magnitude of these estimates because of their observational nature, and our aim in these analyses was to simply confirm the plausible existence of a nonzero relationship in this study cohort. Further work in randomized controlled trials or other study designs that facilitate causal estimates should be pursued to increase our understanding of confounders of this relationship versus intermediaries on the causal path. Summary demographics of this cohort are presented in Table 1.

**Table 1.**
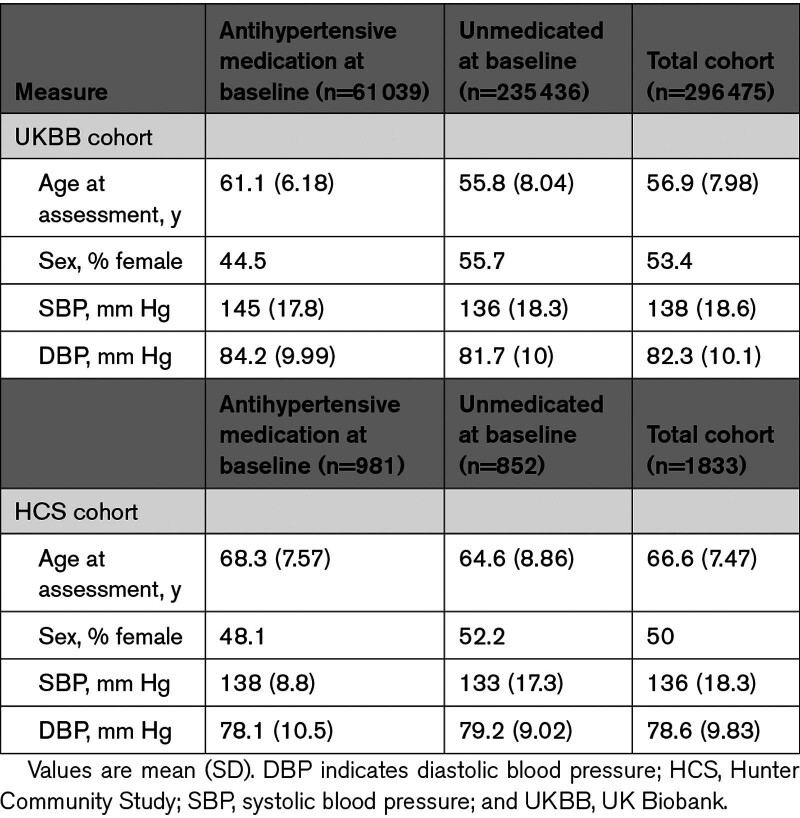
Basic Demographic Characteristics of the UK Biobank and Hunter Community Study Cohorts Used in This Study

### Developing PESs Related to Sodium and Potassium Biology in Hypertension

**Figure 1.**
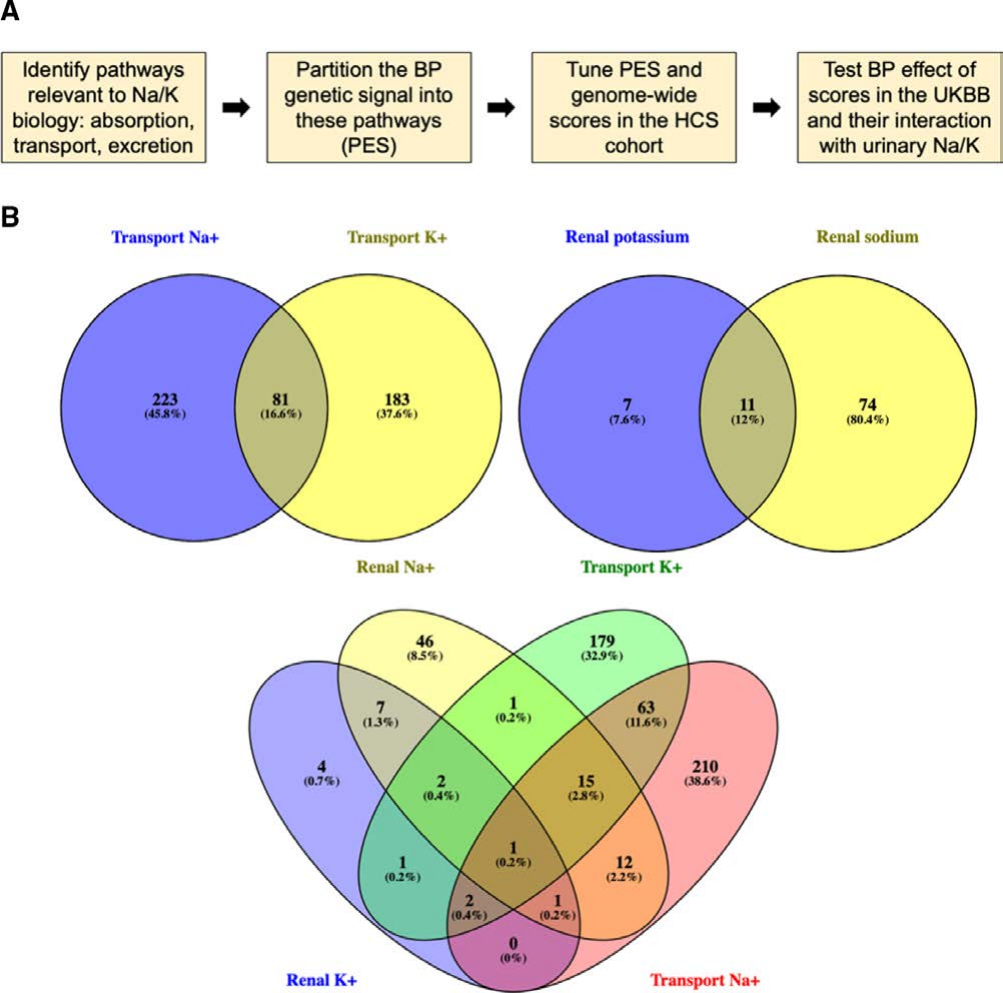
**Development of pharmagenic enrichment scores to inform precision interventions related to sodium and potassium in blood pressure. A**, Condensed overview of the methodology applied in this study to implement the pharmagenic enrichment score (PES) and evaluate its properties. **B**, Venn diagrams depicting the overlap in unique genes between different collated ontologies related to sodium and potassium biology. From left to right: sodium transport genes versus potassium transport genes; renal sodium excretion genes versus renal potassium genes; intersection of the 4 previous gene sets. BP indicates blood pressure; HCS, Hunter Community Study; and UKBB, UK Biobank.

We sought to apply the PES platform to the polygenic signal that influences BP in the context of sodium and potassium biology (Figure 1A). The PES platform theoretically could be deployed in this context to identify individuals who may receive a larger benefit from strategies that reduce sodium and boost potassium, including dietary and drug interventions. Pathways from MSigDB were identified and collated to represent broad areas of sodium/potassium biology: nutrient absorption, transport, and renal excretion (Figure 1B; Tables S1 and S2). Genes were aggregated for the nutrient absorption gene set from 4 different ontological pathways (64 unique genes), whereas the transport and renal excretion gene sets were generated for sodium and potassium individually, and the genes from pathways relevant to each electrolyte were merged. In terms of electrolyte transport, there were 43 sodium transport (304 unique genes) and 37 potassium transport (264 unique genes) pathways, with 487 unique genes once the sodium and potassium gene sets were merged. Renal excretion processes related to sodium yielded 7 pathways (85 unique genes), with 4 pathways for potassium (19 unique genes), and 93 unique genes once the sodium and potassium excretion gene sets were merged. This resulted in 4 distinct gene sets used for PES construction: 1 absorption and 3 each of transport and renal excretion (sodium alone, potassium alone, and sodium and potassium).

The next stage of the genetic component of the study was to tune each of these PES for the optimum variant configuration with SBP and DBP as the outcome, as well as genome-wide PGS. We utilized the HCS cohort for tuning PGS and PES (Table 1), which was split into 2 subsets consisting of participants who self-reported taking an antihypertensive compound and those who did not. First, we profiled genome-wide SBP and DBP PGS at 10 *P* value thresholds for variant inclusion to identify the threshold for each trait that explained the largest mean phenotypic variance across the medicated and unmedicated cohort (Figure S3; Table S4). For SBP, the largest mean variance explained was at the *P*<0.01 threshold (mean Δ *R*^2^=0.01), whereas a lower PGS performed optimally for DBP (*P*_T_<0.005, mean Δ *R*^2^=0.007). PES from 3 overarching biology categories were assessed (nutrient absorption, sodium and potassium transport, sodium and potassium renal excretion), so we selected the best performing score from each of these 3 categories for SBP and DBP, respectively (Table S5). These PESs were as follows: for SBP, absorption (*P*_T_<0.05), sodium and potassium transport (*P*_T_<0.005), and renal sodium and potassium excretion (*P*_T_<0.5); for DBP, absorption (*P*_T_<0.005), sodium and potassium transport (*P*_T_<1), and renal sodium excretion (*P*_T_<1). As a result, we took forward the best PGS and 3 PESs for each of the BP measures at these thresholds and biologic annotation.

The PGS and PES for SBP and DBP, respectively, were then profiled in the full UKBB cohort, although considering only the unmedicated cohort yielded very similar effect sizes (Table S6). When examining the genome-wide PGS, we found that genetically proxied SBP and DBP was associated with a larger effect on SBP and DBP than either UNa^+^ or UK^+^ alone (Table S6). For instance, each SD in SBP PGS was associated with a 1.48 mm Hg (95% CI, 1.42, 1.54) increase in SBP, compared with an association effect size of 1.10 mm Hg (95% CI, 1.03, 1.16) per SD of UNa^+^. The effect size of the SBP PGS among unmedicated participants had a marginally larger point estimate (1.60 mm Hg per SD in PGS) but similar statistical significance as in the full cohort. As visualized in Figure S4, each increasing quintile of SBP PGS exhibits a monotonic relationship with higher mean SBP. We see similar results for DBP PGS but at slightly less statistical significance than SBP. We then tested the relationship of the sodium- and potassium-related PES with measured BP (Figure S5). Each of the tuned PESs were associated with SBP and DBP correcting for 6 tests (*P*<8.33×10^−3^; Table S7). As a sensitivity analysis, these models were repeated with the additional covariate of genome-wide PGS, to control for generalized inflation of the BP genetic signal.^22,24,25^ The transport and renal excretion PES still had nonzero effects on BP that surpassed multiple-testing correction, whereas the absorption PES became only nominally significant (*P*<0.05) upon correcting for PGS, suggesting that score exhibits less population-level relevance for BP once the total polygenic burden is accounted for. The effect sizes of each of these PES on SBP or DBP were also considerably smaller than the full genome-wide common variant architecture as indexed by a PGS, in line with expectation. For instance, each SD in the transport, renal excretion, and absorption SBP PES was associated with a 0.44 mm Hg, 0.28 mm Hg, and 0.16 mm Hg increase in SBP, respectively (PGS unadjusted models), compared with a 1.48 mm Hg increase for PGS. All PES and PGS estimates on SBP and DBP are detailed in Tables S6 and S7. We emphasize the aim of the PES platform is not necessary to identify overall insights of a phenotype that is generalizable to the population; rather, it is designed to be interpreted at an individual level based on their score relative to a population refence. However, PES profiles with statistically significant mean effects at the population level may have more clinical salience for individuals on the upper tail of the distribution.

### Evidence That the Sodium and Potassium Transport PES Exacerbates the Effect of UNa^+^ on BP

We then hypothesized that PESs related to sodium/potassium biology are more likely to display a (polygenic) G×E with measured urinary electrolytes on BP than a biologically undifferentiated genome-wide PGS. This would support the usefulness of the PES approach to identify individuals who may particularly benefit from dietary or pharmacological interventions to lower sodium or raise potassium.

We formally tested this by assessing whether the joint effect of each genetic score (PES or PGS) and the urinary electrolytes on BP significantly departed from additivity in a multiple linear regression model, and thus, constituted evidence for an interaction effect (Table 2). The screening model for G×E effects contained only one interaction term between the genetic score and the urinary electrolyte tested (Table S8), with the G×E estimates and *P* values from the screening models listed in Table 2. The genetic and urinary terms were both standardized to have a mean of 0 and unit variance to aid the interpretability of the interaction terms. For genome-wide PGS, there was nominal evidence of a nonadditive joint effect of SBP PGS and UK^+^ on measured SBP: $\beta_{G\times E}$=−0.071, SE=0.035, and *P*=0.043. The negative sign of this interaction term suggests that the estimated hypertensive effect of each SD in standardized SBP PGS on measured SBP exhibits a small decrease per unit increase in UK^+^ (SD=1). This may indicate that boosting potassium intake could lessen the effect of an underlying genetic propensity for higher SBP; however, this result must be interpreted cautiously. First, the statistical significance is nominal and does not survive correction for the number of tests performed, and the interaction term becomes nonsignificant once G×C (PGS-by-covariate) terms are added in the next modeling stage to control for PGS-by-covariate effects (*P*=0.08). SBP or DBP PGS did not exhibit any other statistically nonzero interactions with UNa^+^, UK^+^, or their ratio. The 6 PES were then screened in the primary G×E model, revealing 2 nominally significant interaction terms for the DBP renal sodium excretion PES with UK^+^ on DBP and the SBP sodium/potassium transport PES with UNa^+^ on SBP. Both interactions remained significant in the G×C models, as well as the final model that also added electrolyte-by-covariate interaction terms (E×C). The estimated effect of UNa^+^ on measured SBP increased per SD increase in the SBP sodium/potassium transport PES ($\beta_{G\times E}$=0.1, SE=0.037, and *P*=8.6×10^−3^), supporting the hypothesis that individuals with a higher PES related to sodium and potassium transport may be particularly susceptible to the hypertensive consequences of sodium intake. This effect was not seen with the PGS for SBP. The interaction between the renal sodium transport DBP PES and UK^+^ was also positive ($\beta_{G\times E}$=0.04, SE=0.02, *P*=0.03). This result may represent a small ablation of the antihypertensive effects of potassium for those with higher PESs in this urinary excretion pathway; in other words, potassium intake may be less effective at lowering BP in those with an elevated urinary excretion PES. Both of the interaction effects should be interpreted cautiously and require replication; however, they represent plausible evidence of G×E.

**Table 2.**
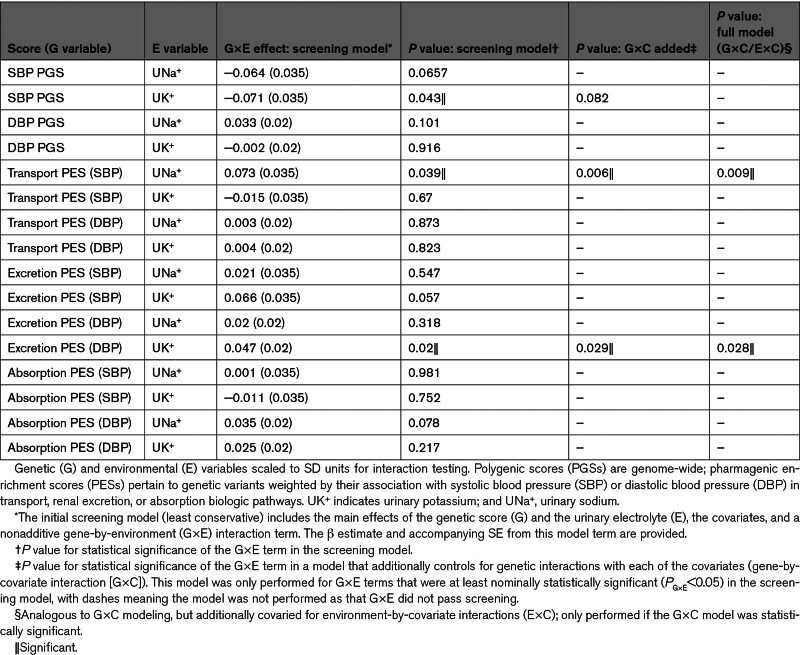
Interaction Testing Between Urinary Electrolytes and Blood Pressure Polygenic Scores or Sodium- and Potassium-Related Pharmagenic Enrichment Scores

As the sodium and potassium transport PES-by-UNa^+^ interaction was most significant, we performed a series of additional sensitivity analyses, described in the following, although we emphasize that the statistical significance of the interaction is nominal. First, we compared the PES-by-sodium interaction with the PGS-by-interaction term directly from the full model and found no evidence that PGS displays an interaction with sodium once G×C and E×C effects are accounted for. This PES also displayed a nonzero positive interaction with a scaled sodium:potassium ratio ($\beta_{G\times E}$=0.1, SE=0.035, *P*=5.6×10^−3^), but not with UK^+^. We then investigated the effect size of UNa^+^ on SBP per decile of the PES versus its effect per decile of the PGS to support the inferred PES-by-sodium interaction (Figure S6; Table S9). This was undertaken as an alternative method to support the existence of the G×E effect without directly estimating the interaction term. As outlined in the Supplemental Material, there is evidence of an inflection point at higher deciles of the Na^+^/K^+^ transport PES in terms of magnitude of the positive correlation between the UNa^+^ and SBP; in other words, the association of the sodium with SBP was larger for participants with higher genetic load in this Na^+^/K^+^ transport pathway, supporting the existence of a G×E effect. For instance, each SD in UNa^+^ was associated with a 1.47–mm Hg (95% CI, 1.24, 1.7) increase in SBP in the top 10% of the PES distribution versus a 0.97–mm Hg (95% CI, 0.75, 1.19) SBP increase in the lowest 10% of the PES distribution. In comparison, the estimated effect of PGS in each of the deciles of UNa^+^ was much more consistent, with not even a trend (*P*<0.1) toward the effect size of UNa^+^ in the highest PGS decile being significantly different than any of the others. We also calculated the variance explained in BP by the Na^+^/K^+^ transport PES for individuals with high (top decile) versus low (bottom decile) UNa^+^. In line with expectation, the proportion of phenotypic variance explained by the PES was very small, as the PES is a subset of the overall polygenic signal. However, the variance explained (*R*^2^) by the PES among individuals with high UNa^+^ (0.15%) was more than triple what was observed for participants in the bottom decile of UNa^+^ (0.04%). An analogous, but smaller, trend was also observed for DBP (0.08% versus 0.02%) in top and bottom deciles of sodium, respectively. We also re-estimated the SEs of this interaction term using HC methods, which did not remove the statistical significance of the departure from additivity (Table S10). Moreover, the interaction remained significant upon adding terms to adjust for BMI and urinary creatinine ($\beta_{G\times E}$=0.1, SE*=*0.04, and *P*=9.95×10^−3^), and there was no evidence for PES-by-BMI or PES-by-creatinine effects. There was also evidence that the variance of SBP was significantly different between quantiles of the PES, thus adding support to the existence of nonadditive effects given that variance-related genetic effects on quantitative traits are enriched for detectable G×E^42,43^ (Supplemental Material). The PES-by-sodium interaction was only nonzero at the optimum threshold after tuning in the HCS (*P*_T_<0.005), with no statistically significant effects upon testing transport PES constructed at the remaining thresholds considered (*P*_T_<1, *P*_T_<0.5, and *P*_T_<0.05), which may indicate the effect is only observable at a less polygenic level of the SBP genetic signal in this pathway. In the full cohort, where individuals taking antihypertensive agents were included and adjusted for by an additional covariate term, the transport PES-by-sodium interaction was directionally consistent but not statistically significant, which may be a product of the power loss because of the variance in SBP being reduced upon adding individuals taking medication.

**Figure 2.**
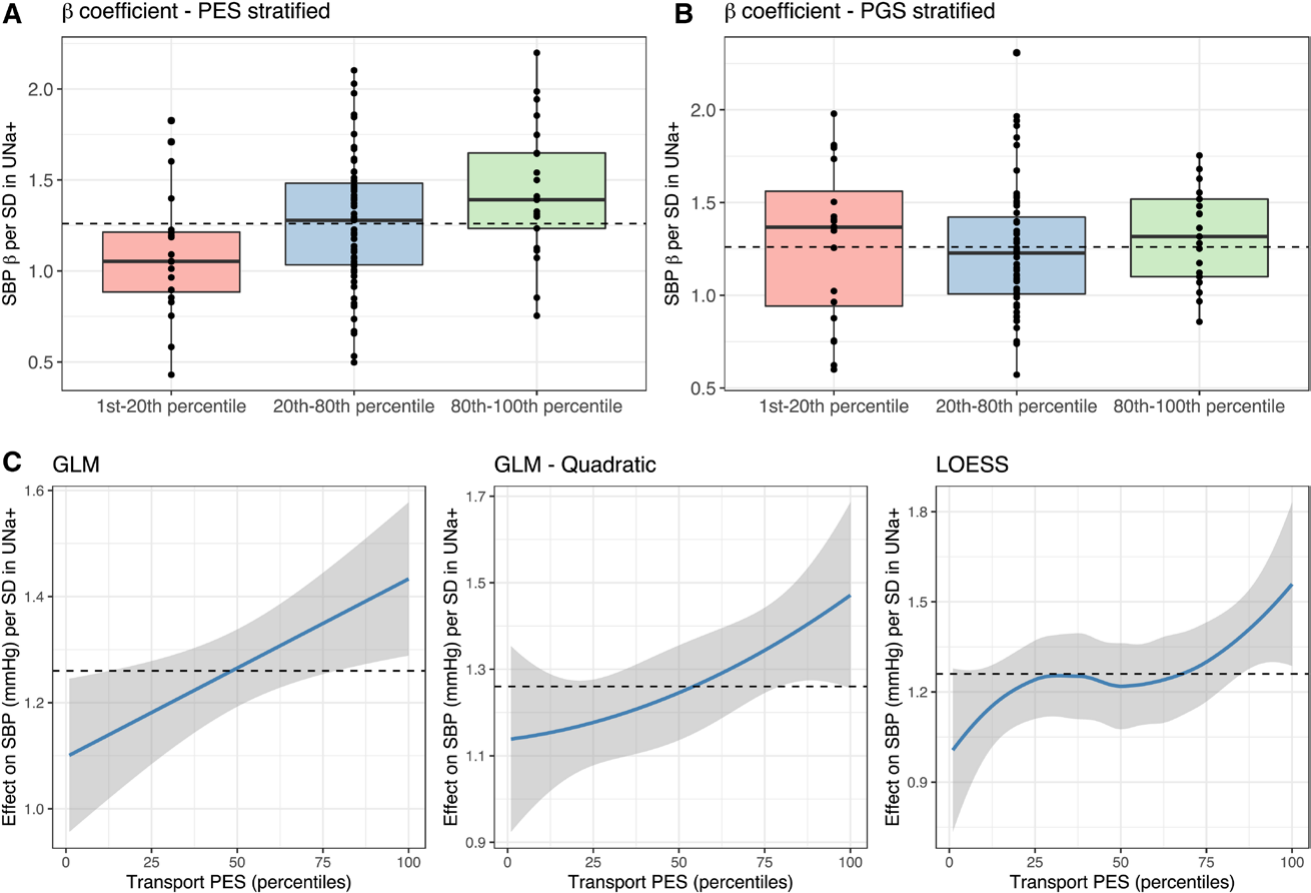
**Association of urinary sodium with systolic blood pressure with increasing sodium/potassium transport pharmagenic enrichment score. A**, The systolic blood pressure (SBP) effect size (mm Hg) per SD of scaled urinary sodium (SD=1) in each percentile of the sodium and potassium transport pharmagenic enrichment score (PES). The per-percentile estimates were grouped as low (1st–20th percentile), moderate (20th–80th percentile), or high (80th–top percentile). Box whisker plots were overlaid on the effect sizes in each group, denoting the median (± interquartile range) urinary sodium (UNa^+^) effect size for each group. The dotted line represents the mean SBP effect size across all percentiles. **B**, Analogous to **A**, but the percentiles instead represent percentiles of genome-wide SBP polygenic score (PGS). **C**, Smoothed trendlines of the effect size of UNa^+^ on SBP in each increasing percentile of the PES. The first panel was smoothed using a generalized linear model (GLM), the second panel adds a quadratic term, and the third panel uses local polynomial regression (locally estimated scatterplot smoothing [LOESS]). All panels demonstrate the larger effect size association of urinary sodium with SBP at elevated PES.

We explored the relationship between genetic risk as indexed by the Na^+^/K^+^ transport PES or genome-wide PGS and the association of UNa^+^ with SBP in greater resolution by splitting the cohort into percentiles of each genetic score. We found that there was a statistically significant relationship between increasing percentile of the Na^+^/K^+^ transport PES and the effect size of the UNa^+^ association with SBP in that percentile (*P*=9.52×10^−3^), which was not seen for genome-wide PGS (Figure 2A and 2B). This modification of the UNa^+^/SBP correlation by increasing Na^+^/K^+^ transport PES was clear upon fitting a linear curve, considering quadratic effects, or using local polynomial regression (Figure 2B). These analyses were also performed for the other PES that was significant in the full model (renal transport DBP PES), further supporting that elevated genetic risk in that pathway appeared to lessen the negative association of UK^+^ with DBP (Figure S7).

We found evidence that variants comprising the PES physically annotated to genes in the sodium/potassium transport gene set were functionally relevant to those genes. This was achieved through correlating the PES with mRNA expression in blood in an independent genotyped sample (Methods in the Supplemental Material; Figure S8).^52^ We found that PES had larger mean absolute effects on mRNA expression of genes within the sodium/potassium transport gene set (*t*[286]=4.56 and *P*=7.72×10^−6^) than that of genome-wide PGS, supporting the functional relevance of variants in the PES to those genes.

### Interaction Analyses Considering Renal Function and Urinary Dilution

The use of spot urinary electrolyte samples may be confounded by factors including renal function and urinary dilution effects. As a result, we explored whether the observed interaction between the Na^+^/K^+^ transport PES and UNa^+^ on SBP may be affected by these factors. We first demonstrated that repeating the percentile analysis shown in Figure 2 adjusted for eGFR produced highly concordant results (*r* = 0.97; Figure S9). Splitting the cohort into individuals with eGFR ≥90 mL·min·1.73 m^2^ or <90 mL·min·1.73 m^2^ also did not alter the G×E estimate to a statistically significant extent (Supplemental Material; Figure S10). We then modeled UNa^+^ to urinary creatinine ratio (UNa^+^:UCr) in the G×E model. The use of the UNa^+^:UCr phenotype in a genetic study is more challenging because of bias that can be induced through adjustment of heritable covariates, potential nonlinear relationships between the sodium and creatinine, as well as heterogeneity induced by influences on UCr that are not related to renal physiology, such as muscle mass.^53,54^ Despite this, the interaction between UNa^+^:UCr and the Na^+^/K^+^ transport PES remained similar in magnitude and statistically significant ($\beta_{G\times E}$=0.089, SE=0.04, *P*=0.042; full model G×C and E×C terms added). We also repeated the percentile modeling using UNa^+^:UCr and found moderate, statistically significant, per-percentile concordance with the raw UNa^+^ estimates. More extensive explanation of the UNa^+^:UCr analyses, as well as the statistical and technical considerations of using a ratio term for G×E tests, can be found in the Supplemental Material (Figures S11 and S12).

### Examining the Interplay Between Overall Diet Quality and Genetic Propensity for Higher BP

We used the 24-hour recall RFS as an index of diet quality at time of baseline assessment in the UKBB.^50^ After harmonizing with our study cohort and removing any individuals who self-reported they did not eat their usual diet yesterday, there were 37 357 participants with genetics, BP, electrolytes, and sufficient 24-hour recall responses to calculate the RFS (Table S11). The mean RFS was 7.4 (SD=2.99) and ranged from 0 to 20. Including medicated individuals, an RFS was correlated with lower SBP, DBP, UNa^+^, and UNa^+^/UK^+^ ratio (Table S12). These correlations remained similar when only considering participants not taking antihypertensives (Table S12). We observed a small negative relationship between RFS and UK^+^ regardless of medication status, and even after covarying for total energy intake. Estimated potassium intake from the dietary questionnaire, however, was positively correlated with UK^+^, in line with expectation. Sodium intake in this UKBB subset was also correlated with UNa^+^; for instance, self-reported addition of salt to food (usually or always) was associated with a 0.236 SD (95% CI, 0.208, 0.265) increase in UNa^+^ relative to individuals who answered sometimes or never. Given that UK^+^ is known to be elevated by improved diet quality, and the observed relationship with the RFS is small, we suggest this could be driven by another unaccounted-for variable, although the RFS should be further investigated for validation purposes in terms of its relationship with urinary electrolytes. In Figure S13, the distribution of the urinary electrolytes, as well as estimated K^+^ intake, per quartile of the RFS is visualized. This consolidates that the relationship between UK^+^ and RFS is less marked than the other 3 variables.

We then tested whether any of the genetic scores that demonstrated at least nominal evidence for an interaction with the urinary electrolytes (SBP PGS, Na^+^/K^+^ transport SBP PES, renal excretion DBP PES) also exhibited a nonadditive joint effect with diet quality on BP. In the baseline G×E model, there was nominal evidence that increasing diet quality lessened the hypertensive effects of the SBP PGS on SBP ($\beta_{G\times E}$=−0.19, SE=0.097, and *P*=0.046), which was similar to its joint effect with UK^+^. This interaction was not statistically significant once the full G×E model was specified (G×E and ExC effects included); however, we are likely underpowered in this smaller cohort to detect nonadditive effects. The PES did not demonstrate any evidence of interaction with diet quality on BP, which suggests their relevance is more directly related to sodium and potassium. As an exploratory analysis, we split the dietary cohort into quartiles of the RFS and tested whether the G×E effect between the Na/K transport PES and UNa^+^ was larger at lower diet quality but did not find any statistically significant evidence to that effect. Larger sample sizes with dietary data available will likely be required to fully appreciate the moderating effects of diet quality.

## DISCUSSION

In this study, we explored whether genetics could be used to overcome response heterogeneity by identifying individuals more likely to respond to interventions to lower BP related to sodium and potassium. Hypertension is regarded as an especially pressing public health issue, particularly because many individuals with hypertension remain undiagnosed, and response among those who do receive care is often suboptimal.^55^ Shifting sodium and potassium consumption toward lower sodium and higher potassium levels is regarded as an important dietary intervention for both prevention and management, although significant heterogeneity in response is observed.^15,17^ We demonstrated how PGSs for BP tailored to sodium and potassium biology could be used to identify individuals who may receive a greater benefit from sodium- and potassium-related interventions, although, given the flexibility of the PES, this could also be extended practically to any other dietary or pharmacologic intervention for hypertension. We uncovered that those scoring highly on PES constructed among pathways related to sodium and potassium may experience exacerbated hypertensive effects of sodium, as well as sodium/potassium ratio, on systolic BP. This nonadditive joint effect suggests the existence of G×E, which would support the promise of the PES platform to identify optimal responders to interventions that reduce sodium. The same interplay with sodium was not seen for a traditional genome-wide PGS, although there was comparatively more nominal evidence that the effect of generalized genetic risk (PGS) was lessened by increased potassium and overall diet quality as expressed by the RFS. In other words, the sodium/potassium transport PES appears to be more specifically related to the interplay between urinary electrolytes and BP, revealing individuals for whom sodium appeared to exhibit larger hypertensive effects. PGS, as a genome-wide score, is informative for overall risk prediction, but does not appear to exhibit this same biological specificity in relation to sodium or potassium.

Several important limitations and caveats should be considered when interpreting the results of this study. First, whereas BP is significantly heritable, genome-wide PGS for BP only explain a small amount of phenotypic variance, although it is larger than that of the individual urinary electrolytes. We predict that the scores would likely be improved using a larger GWAS, such as recent ones that include the UKBB; however, we avoided these combined summary statistics (and the HCS data) to maintain independence from the discovery cohort to minimize the possibility of overfitting. Whereas we leveraged data from individuals with European ancestry in this study, which afforded the greatest sample size and power, to translate these findings equitably, the portability of these scores to individuals from different races and ethnicities and admixed individuals should be investigated. Transancestral GWAS for BP are becoming increasingly well-powered, which will likely be an important resource for these efforts. There is emerging evidence that the BP genetic signal derived from European populations may have relatively high transferability across some ancestries,^56^ but not others.^57^ We also caution that the inferred G×E effect between UNa^+^ and the sodium/potassium transport PES is only nominal in terms of statistical significance, although the estimate and inferred G×E effect was stable to the various sensitivity analyses performed. Simulation approaches in follow-up work should be developed to better understand the null expectation for G×E effects when applied to partitioned scores such as the PES. Future replication in different data sets will also be important, ideally coupled with 24-hour urine collection as a biomarker of sodium and potassium intake. We justify our use of spot urinary electrolytes in this study because we had a large sample size and the use of heritable covariates such as BMI in equations to estimate 24-hour excretion from spot samples is a potential source of bias in genetic studies if genetic effects are mediated through the covariate.^53^ However, in our additional sensitivity analyses that investigated the influence of eGFR and urinary dilution through indexing to urinary creatinine, we found evidence that the inferred G×E effect remains significant. Leveraging 24-hour urine samples will allow more accurate effect sizes to be estimated in future work. In a similar vein, it is not inherently clear from this study whether the estimated G×E effect sizes are clinically significant despite their statistical significance. This will need to be resolved in intervention studies, for which we discuss some recommendations in the next section. We also assumed upon constructing the PES that the variants physically annotated to sodium- or potassium-related genes would exert their effects on those same genes, although because of the extent and complexity of linkage disequilibrium in the human genome, this may not necessarily be the case. Curated gene-ontology pathways were also used to identify the genes for which to construct the PES; however, these genes should also be subject to more expansive examination to identify the most salient genes related to sodium and potassium biology. Some more general discussions of limitations of the PES approach have been extensively discussed in previous work.^22,23,26^ In addition, we assume linearity in individual relationships between both the urinary electrolytes and the genetic scores with BP. There is some observational evidence to support a nonlinear relationship between sodium and potassium intake and BP,^17^ although this is far from settled. In terms of genetics, a linear or additive model of common genetic variation at the population level is the model supported by the majority of empirical evidence. However, we see some evidence of nonlinear genetic correlates through differences in phenotypic variance associated with the PES, which, in line with previous work, were smaller than the mean effects.^43^ It is important to make the distinction in considering these matters between nonadditive effects of genetic variants versus nonadditive joint effects between genetics and environmental variables, which is a G×E effect.

Follow-up studies could advance this work. Although there are limitations to the results of this study, as outlined, facilitating dietary strategies to obtain a healthy sodium-to-potassium ratio managed by health professionals is a low-risk intervention that could be readily investigated in terms of its modification by genetics. Total genetic risk for hypertension (PGS) may be a useful approach for risk stratification and individuals with high genome-wide genetic risk load may benefit from overall strategies to improve diet quality, with some weak evidence to this effect seen in these data through the RFS-by-PGS interaction. Using PGS to stratify studies of improving diet quality to manage BP retrospectively or prospectively would be advantageous to understand its usefulness. Whereas total genetic risk may be a useful marker to inform general dietary interventions, it likely lacks the biological specificity to tailor interventions related to specific nutrients or dietary patterns. Therefore, the PES framework oriented around biology related to more specific variables, such as sodium, may be more effective to identify individuals who will respond more readily to more targeted interventions. Given that genotyping and associated processing costs are rapidly decreasing, this could be a cost-effective solution if proven to be effective. The effectiveness of the PES to identify optimal responders to low-sodium diets could be evaluated through randomized controlled trials or intervention studies stratified by the PES. Another important future direction is to better understand consumer and clinician attitudes to the use of genetics to inform dietary interventions in hypertension. One advantage of the PES in this regard is that, because the PES is not specifically designed to capture overall risk, it would likely be communicated in a similar vein to existing companion diagnostics that leverage genomics, rather than as a metric of risk for a phenotype. However, input from genetic counselors and other key stakeholders will be important to ensure that any information regarding genetics is delivered in a way that minimizes harm. Our pilot observational data support the promise of genetics to inform dietary interventions in hypertension with greater precision.

## ARTICLE INFORMATION

### Sources of Funding

Dietary data processing was funded by a pilot grant from the School of Health Sciences, The University of Newcastle (Dr Clarke). Dr Collins was supported by an investigator grant from the National Health and Medical Research Council. Dr Cairns was supported by a National Health and Medical Research Council, Senior Research Fellowship and Grants.

### Disclosures

Drs Reay and Cairns have filed a patent related to the use of the pharmagenic enrichment score framework in complex disorders (WIPO patent application WO/2020/237314), an approach used in a section of this study. Dr Cairns is director of a company that aims to commercialize the pharmagenic enrichment score platform (PolygenRx Pty Ltd), in which Dr Reay holds equity. The remaining authors declare no competing financial interests.

### Supplemental Material

Methods

Results

Figures S1–S13

Tables S1–S12

References 58–61

## Supplementary Material
