# Supplementary material for: Using Genetics to Inform Interventions Related to Sodium and Potassium in Hypertension
Source: Circulation. 2023 Dec 21;149(13):1019–32. doi: 10.1161/CIRCULATIONAHA.123.065394 (PMC10962430; doi:10.1161/CIRCULATIONAHA.123.065394)
Supplement: Supplementary file 1 [file cir-149-1019-s001.pdf]

SUPPLEMENTAL MATERIAL: Utilising genetics to inform interventions related to sodium and potassium in hypertension

William R. Reay[1,2], Erin Clarke[3,4], Shaun Eslick[3], Carlos Riveros[5], Elizabeth G. Holliday[5,6] Mark A. McEvoy[7], Roseanne Peel[6], Stephen Hancock[6], Rodney J. Scott[1,8], John R. Attia[5,6], Clare E. Collins[3,4†*], Murray J. Cairns[1,2†*]

[1]School of Biomedical Sciences and Pharmacy, The University of Newcastle, Callaghan, NSW, Australia

[2]Precision Medicine Research Program, Hunter Medical Research Institute, New Lambton, NSW, Australia

[3]School of Health Sciences, The University of Newcastle, Callaghan, NSW, Australia

[4]Food and Nutrition Research Program, Hunter Medical Research Institute, New Lambton, NSW, Australia

[5]Hunter Medical Research Institute, New Lambton, NSW, Australia

[6]School of Medicine and Public Health, The University of Newcastle, Callaghan, NSW, Australia

[7]Rural Health School, La Trobe University, Bendigo, Victoria, Australia

[8]Cancer Detection and Therapy Research Program, Hunter Medical Research Institute, New Lambton, NSW, Australia

SUPPLEMENTAL MATERIAL

Supplementary Methods

Supplementary Results

Supplementary Figures 1-13

SUPPLEMENTARY METHODS

UK Biobank (UKBB) Quality Control

In the UKBB, 13,568,914 autosomal variants survived a series of quality control steps, including, imputation quality filtering (INFO > 0.8), minor allele frequency (MAF) > 1×10^{-4}, call rate > 0.98, and filtration of strong deviations from the Hardy–Weinberg equilibrium. We performed analyses for this pilot study in the largest ancestral group in the UKBB to maximise power (White British), however, we acknowledge the need for follow up studies to assess the transferability of our findings to other ancestral populations to ensure equitable development and application of genetics research.

Polygenic score generation (PGS) and tuning

PGS in individual i sums the effect size of j variants from the GWAS on blood pressure ($\hat{\beta}_j$), multiplied by its allelic dosage under an additive model ($G_{ij} \epsilon \{0, 1,2\}$, equation 1). The full set of M variants included in the score is selected by linkage disequilibrium clumping and thresholding (LD C+T), whereby SNPs are 'clumped' such that the retained SNPs are largely independent and 'thresholded' based on their association P value in the GWAS[58].

$$PGS_i = \sum_{j=1}^{M} \hat{\beta}_j G_{ij} \quad (1)$$

The 1000 genomes phase 3 European reference panel was utilised to perform the LD C+T via a plink v1.9 wrapper implemented by the R ieugwasr package v0.1.5[31,32]. We selected $r^2 = 0.1$ for clumping in 250 kilobase chunks, with PGS weights at each of the following P value thresholds generated for subsequent tuning (P_T): 5×10^{-8}, 1×10^{-5}, 1×10^{-4}, 5×10^{-3}, 1×10^{-3}, 0.01, 0.05, 0.1, 0.5, 1. The summary statistics were cleaned (variants with minor allele frequency < 0.01 duplicated variants, strand ambiguous variants (A/T, T/A, G/C, and C/G), and variants tested in < 90% of the sample all removed), as well as variants within the extended major histocompatibility complex (MHC) on chromosome 6 due to its LD complexity.

Pharmagenic enrichment score (PES) generation and tuning

Variants annotated to sodium/potassium biology were then subjected to LD C+T, T ϵ {0.005, 0.05, 0.5, 1}. As outlined in previous work, these thresholds in subsetted scores like PES are designed to capture differing levels of the polygenic signal whilst still retaining enough independent variants such that they are adequately informative of the biology of the

pathway[22,24-25]. After this process, PES are profiled in the same fashion as genome wide PGS, but M consists of only clumped variants within the gene-set of interest (equation 2).

$$PES_i = \sum_{j=1}^{M} \hat{\beta}_j G_{ij} \quad (2)$$

Profiling and tuning in the HCS was identical to the PGS.

Gene-by-environment effect modelling

We firstly screened the best performing PGS and PES for SBP and DBP in a model that added an interaction term between the urinary electrolyte and the genetic score via specified in equation 3, which includes only a GxE term ($\beta_{G\times E} G_i E_i$).

$$y_i \sim \beta_0 + \beta_C C_i' + \beta_E E_i + \beta_G G_i + \beta_{G\times E} G_i E_i + \varepsilon_i \quad (3)$$

PES or PGS with a nominally significant ($P < 0.05$) GxE term with either urinary sodium or potassium were the carried forward for additional sensitivity analyses. As shown previously, spurious GxE effects can be detected when interaction terms between the environmental exposure of interest (urinary sodium or potassium) are not included with all covariates ($\beta_{C\times E} C_i' E_i$), as well as interaction terms between the genetic term and all covariates ($\beta_{G\times C} G_i C_i'$)[37]. To address this we, we constructed two additional models for GxE pairs with some evidence for non-additivity that controlled for gene-by-covariate (GxC) effects (equation 4), and both GxC and covariate-by-environment (CxE) effects (equation 5).

$$y_i \sim \beta_0 + \beta_C C_i' + \beta_E E_i + \beta_G G_i + \beta_{G\times E} G_i E_i + \beta_{G\times C} G_i C_i' + \varepsilon_i \quad (4)$$

$$y_i \sim \beta_0 + \beta_C C_i' + \beta_E E_i + \beta_G G_i + \beta_{G\times E} G_i E_i + \beta_{G\times C} G_i C_i' + \beta_{C\times E} C_i' E_i + \varepsilon_i \quad (5)$$

For the most significant GxE effect detected in both models we performed additional sensitivity analyses. Firstly, we estimated the effect of the urinary sodium on blood pressure at each decile of either the PES or PGS to screen for evidence that the effect size does not monotonically increase or decrease at differing levels of the environmental exposures. The slopes of the E effect amongst participants in the top decile ($\hat{\beta}_{10}$) vs each other decile ($\hat{\beta}_k$) were then sequentially tested for statistically significant differences (equation 6).

$$Z = \frac{(\hat{\beta}_{10} - \hat{\beta}_k)}{\sqrt{(SE_{10}^2 + SE_k^2)}} \quad (6)$$

We also tested the effect of adjusting for urinary creatinine, as well as estimating interactions in the full cohort that covaries for medication status. The statistical significance of GxE effects are also particularly prone to inflation due to heteroskedasticity[39], and as such, we re-estimated the standard errors as heteroskedasticity consistent (HC) standard errors, specifically leveraging the HC0 (White's Estimator) and HC3 methods via the sandwich R package v3.0-

1[40,41]. Finally, we also considered whether the *G* term of interest (PGS or PES) was associated with differences in blood pressure variance, rather than just mean effects. Genetic correlates with the variance of quantitative traits have previously been shown to be enriched for factors that display detectable GxE effects[42,43]. We tested this by splitting the relevant score into quantiles followed by testing for significant differences in variance between these quantiles using Levene's test. Specifically, this involved the cohort being split into both quartiles and deciles for comparison of variance using Levene's test. A stringent normalisation approach was used for this testing of variance effects, in accordance with previous literature related to genetic correlates of variance[43], whereby the residuals of a model that regressed age, age^2, 20 principal components, assessment centre, and assessment month on blood pressure were winsorized at five standard deviations above or below the mean, followed by normalisation to have a mean of zero and unit variance. This model was constructed in males and females separately to remove mean and variance differences between sexes.

Exploring the effects of estimated glomerular filtration rate (eGFR) and indexing to urinary creatinine

We sought to investigate the influence of renal function and urinary dilution effects on our interaction results. Indexing to urinary creatinine is undertaken in some studies to attempt to account for interindividual variation in urinary dilution that could impact the interpretation of spot urinary biomarkers[59]. We re-estimated the GxE effect of the sodium/potassium transport PES with urinary creatinine:urinary sodium (UCr:UNa$^+$) in the full model that included both GxC and ExC effects. We also repeated the percentile analyses with UCr:UNa$^+$ as the exposure variable and assessed the correlation of the estimated effect sizes with that of raw urinary sodium. Two different equations were then used to estimate GFR in each participant in our UKBB study cohort. We decided to use serum cystatin C as the main input for the eGFR equations given we had already performed creatinine related analyses and to explore the effects of different biomarkers. The eGFR equations were implemented using the *nephro* v 1.3 R package. Specifically, these were the CKD-EPI equation for cystatin C proposed by Inker *et al.* and the age and sex weighted equation for cystatin C proposed by Stevens *et al.*[44,45]. In line with previous genetic studies using eGFR, we winsorized eGFR values below 15 ml/min/1.73m^2 or above 200 ml/min/1.73m^2 – resulting in the exclusion of 41 participants[60]. We re-estimated the per-percentile effects additionally adjusting for eGFR, performing these analyses for both equations separately. We also split the cohort into an eGFR above or below 90 ml/min/1.73m^2 and estimated the full model GxE effect in each partition. The value of 90

ml/min/1.73m^2 is somewhat arbitrary but is often used as a value to represent 'normal' kidney function. These effect sizes were formally compared for statistically significant difference using the *Z* test described above in equation 6. Finally, we also included eGFR as a continuous variable in the full GxE model (ExC and GxC included).

Transcriptomic correlates of the polygenic scores

We investigated the similarity between the gene expression signature (correlation with mRNA expression) associated with the SBP sodium/potassium transport PES and genome wide SBP PGS, respectively. The Genotype-Tissue Expression (GTEx) v8 post-mortem dataset was utilised for this purpose, with the use of these data approved through dbGaP (application ID = #1017132-1, project ID = #27869)[52]. The transport SBP PES and genome wide SBP PGS were profiled in 838 GTEx individuals sequenced with whole-genome sequencing (WGS) after an extensive quality control (QC) pipeline performed by GTEx, described in detail elsewhere[52], yielding 46,569,704 variants. Of the GTEx tissues available, we focused on whole blood mRNA expression captured by RNA sequencing (RNA-seq) as an exploratory analysis as it is one of the most well-powered tissues in the dataset. Specifically, the effect of these scores was estimated on each transcript drawn from the matrix of normalised expression of 20,247 transcripts amongst 558 individuals with inferred homogenous genetic ancestry that was generated by GTEx for quantitative trait loci estimation (QTL). As outlined previously by GTEx[52], RNA-seq reads after alignment and initial QC were normalised between samples, lowly expressed genes removed, and expression values subjected to inverse-rank normal transformation across samples. Probabilistic Estimation of Expression Residuals (PEER) normalisation was then applied to account for hidden batch effects and other forms of excessive technical or biological variance via estimation of latent covariates (termed PEER factors)[61]. In line with the GTEx QTL pipeline, we regressed the PES and PGS separately on each transcript in a linear model covaried for donor sex, five SNP-derived PCs, 15 PEER factors, WGS library preparation protocol (PCR-free or PCR-based), and WGS platform (Illumina HiSeq 2000 or Illumina HiSeqX). The correlation between the regression *t* values (beta/SE) for each transcript with PES or PGS as the explanatory variable, respectively, was tested using linear regression. We estimated this correlation across the entire blood transcriptome and specifically within the genes from the sodium/potassium transport pathway used to construct the PES that were detectable in this dataset. The mean difference in these mRNA signatures between the scores was then tested using a paired *t* test.

SUPPLEMENTARY RESULTS

Association of urinary sodium per decile of the sodium/potassium transport PES

We formally compared the urinary sodium effect sizes on SBP in each decile of the PES individually relative to the highest decile and found the hypertensive effect of sodium in the highest decile was statistically significantly larger ($P < 0.05$) relative to the first, second and fifth decile, with a trend ($P < 0.1$) for the third and seventh decile (Table S9).

Differential variance between quantiles of the PES

There was also evidence that the variance of SBP was significantly different between quantiles of the PES, thus adding support to the existence of non-additive effects given variance related genetic effects on quantitative traits are enriched for detectable GxE[42,43]. This phenomenon of unequal SBP variance was more pronounced between quartiles ($P = 5.67 \times 10^{-3}$, Levene's test), than deciles ($P = 0.049$, Levene's test) of the transport PES.

Exploring the effect of eGFR

Firstly, we re-performed the percentile analyses whereby the effect of urinary sodium on SBP was estimated in each percentile of the sodium/potassium transport PES. In this instance, we additionally added a covariate of eGFR (Stevens *et al.* and CKD-Epi equations both evaluated in separate models). Analogous to the eGFR unadjusted results, we found a significant association between increasing PES percentile and the urinary sodium effect size that was not seen for genome-wide PGS. The effect sizes between the unadjusted and adjusted analyses were also highly concordant ($r = 0.973$ – Stevens *et al.* eGFR, $r = 0.972$ – CKD-Epi). We then added eGFR to the GxE model, inclusive of eGFR-by-urinary sodium and eGFR-by-PES coefficients. Our GxE estimate remained quite consistent and statistically significant in these models – CKD-Epi eGFR: $\beta = 0.08$, $SE = 0.04$, $P = 0.042$, Stevens *et al.* eGFR: $\beta = 0.08$, $SE = 0.04$, $P = 0.041$. Splitting the cohort into eGFR below or above 90 ml/min/1.73m^2 yielded GxE estimates that were not statistically different from each other as compared using the Z test outlined in equation 6 ($P = 0.622$). The magnitude of the GxE effect was larger in those with eGFR < 90 ml/min/1.73m^2, however, considering the associated standard error this in fact was not significantly different than those ≥ 90 ml/min/1.73m^2. The PES was then split into deciles in each of these two partitions to estimate the per-decile urinary sodium effect size. We found a very similar pattern of a generally increasing urinary sodium effect size at elevated PES

(Figure S10). We do note that the GxE effect was in either eGFR subset less precise than in the full cohort, which is likely a product of a reduced sample through partitioning by eGFR. However, this does reinforce the need for larger samples in future work such that these effects can be investigated with greater fidelity.

Benchmarking to urinary creatinine

We found that upon benchmarking urinary sodium to urinary creatinine (UNa^+:UCr) that the GxE effect with the sodium/potassium transport PES remained statistically significant, although was marginally attenuated relative to the raw urinary sodium estimate. Upon using sandwich estimators (HC) to account for heteroskedasticity, the standard error increases from approximately 0.044 to 0.0516, and thus, decreasing statistical significance to a trend (P = 0.08). We sought to investigate this further by repeating the PES percentile modelling with UNa^+:UCr. There was moderate concordance between the per-percentile effect sizes of UNa^+:UCr vs UNa^+ on SBP ($r = 0.324$, $P = 1.01 \times 10^{-3}$). As visualised in figure S11, there is still clear evidence that the association of UNa^+:UCr is smaller for individuals with low PES < 20th percentile. However, there is much more heterogeneity above that threshold, with no consistent evidence of a difference between moderate (20th to 80th percentile) and high PES (> 80th percentile). This can also be seen using the smoothed curves (LOESS, Figure S11) – whilst the attenuated association with SBP in the low PES group is relatively consistent for both UNa^+:UCr and UNa^+, the increasing SBP effect size beyond that is comparatively more linear with raw UNa^+. This perhaps is not surprising in the sense that UNa^+:UCr is a less interpretable phenotype than raw UNa^+ in the context of genetics as it assumes a purely linear relationship between UNa^+ and UCr – which are two heritable traits for which genetic variants plausibly will act differently on - as well as inducing effects of creatinine unrelated to renal function like body and muscle mass. In summary, we still see evidence of a gene-by-environment effect using UNa^+:UCr, particularly for individuals with low PES; however, it is weakened compared to raw UNa^+. Given the limitations of benchmarking to creatinine, future work should be focused on testing this interaction with 24-hour urine. For instance, as visualised in Figure S12, there is evidence of a non-linear relationship between urinary creatinine and urinary sodium at higher creatinine values > 1 SD above the mean. The data presented herein does at least suggest in the interim that the non-additive interplay between the PES and sodium is not purely an artefact of factors such as urinary dilution.

SUPPLEMENTARY FIGURES

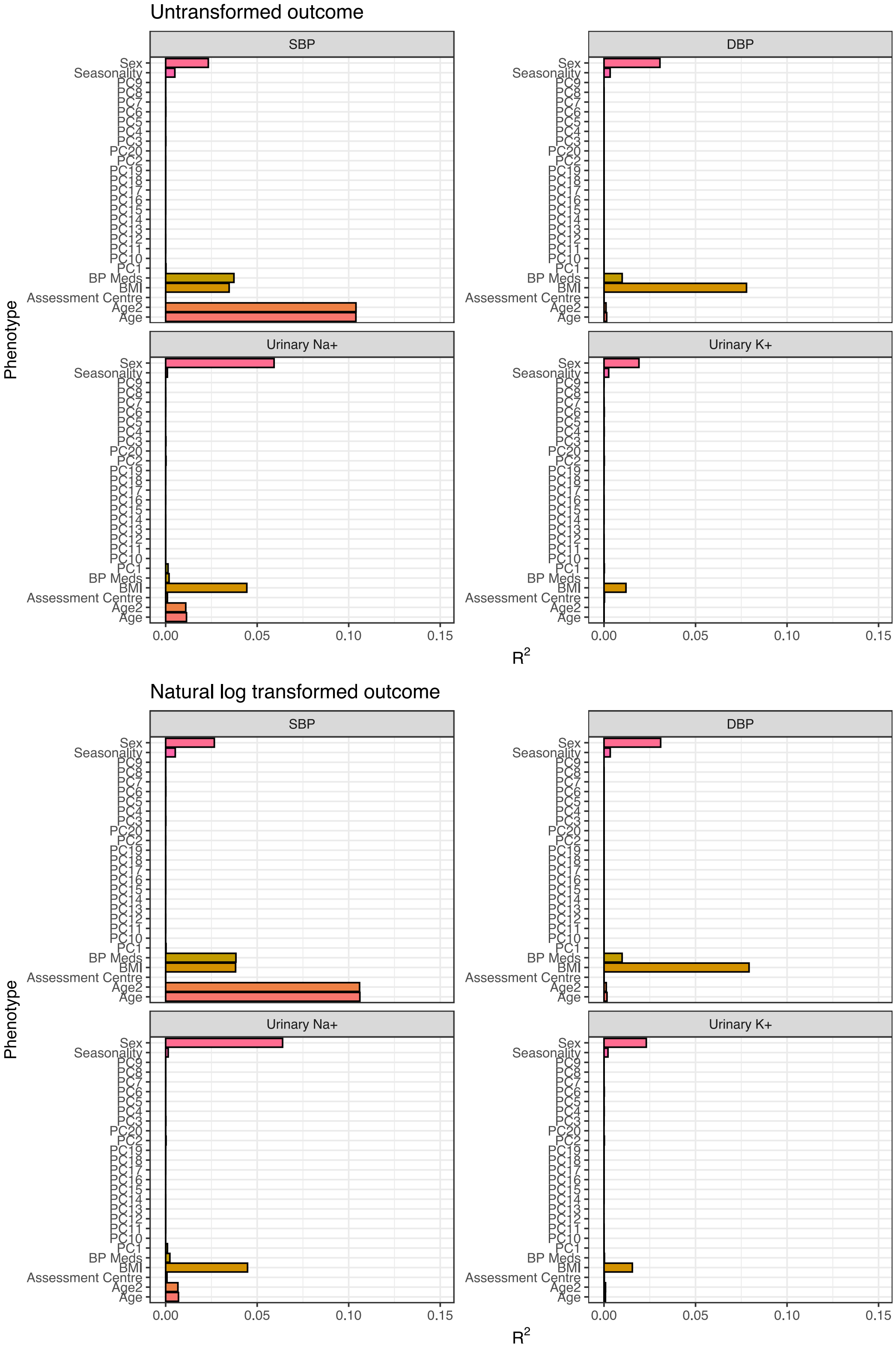

Figure S1. **Sources of variation in blood pressure and urinary electrolytes in the UK Biobank study cohort**. Variance explained (R^2) by each of the variables on the y-axis for systolic blood pressure (SBP), diastolic blood pressure (DBP), urinary

sodium (urinary Na^+), and urinary potassium (urinary K^+) from separate linear models. The outcomes on the left are untransformed, whilst the righthand plots represent results from models wherein the outcome was natural log transformed.

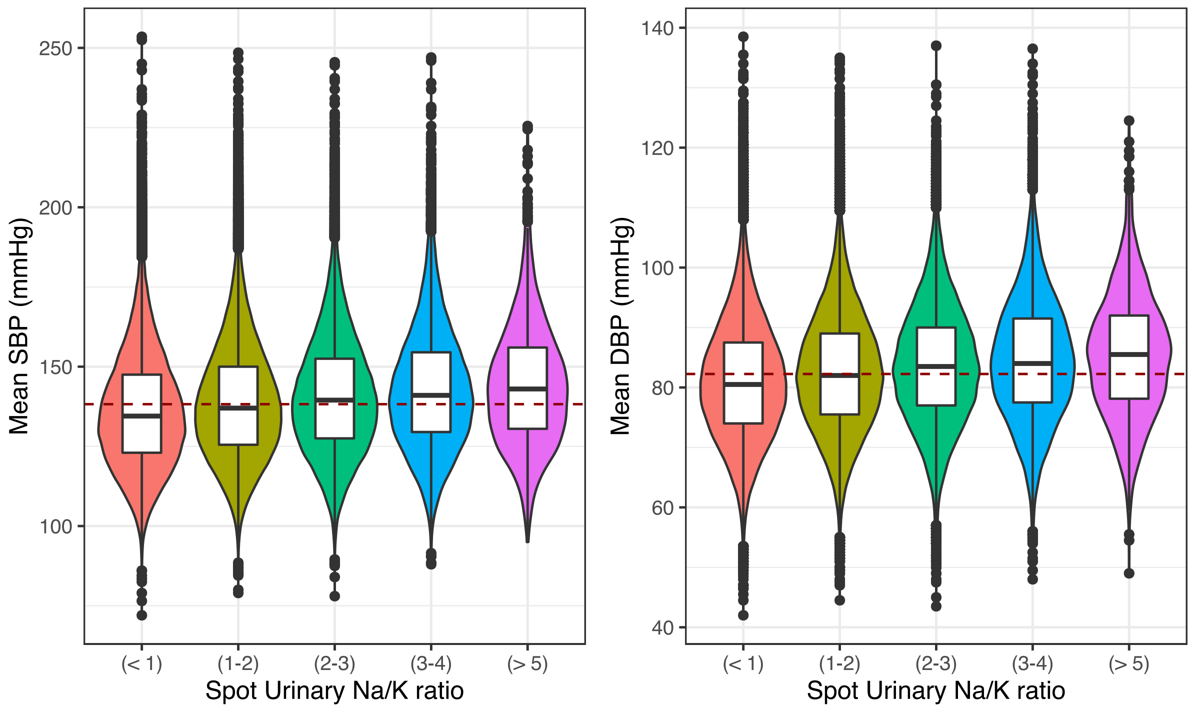

Figure S2. **Relationship between increasing urinary sodium/potassium ratio and blood pressure.** Systolic blood pressure (SBP, left) and diastolic blood pressure (DBP, right) is plotted for categories for spot urinary sodium potassium ratio as a box-and-whisker plot overlaid on a violin plot. The x-axis denotes a sodium/potassium ratio < 1, between 1 and 2, between 2-3, between 3-4, and greater than 5.

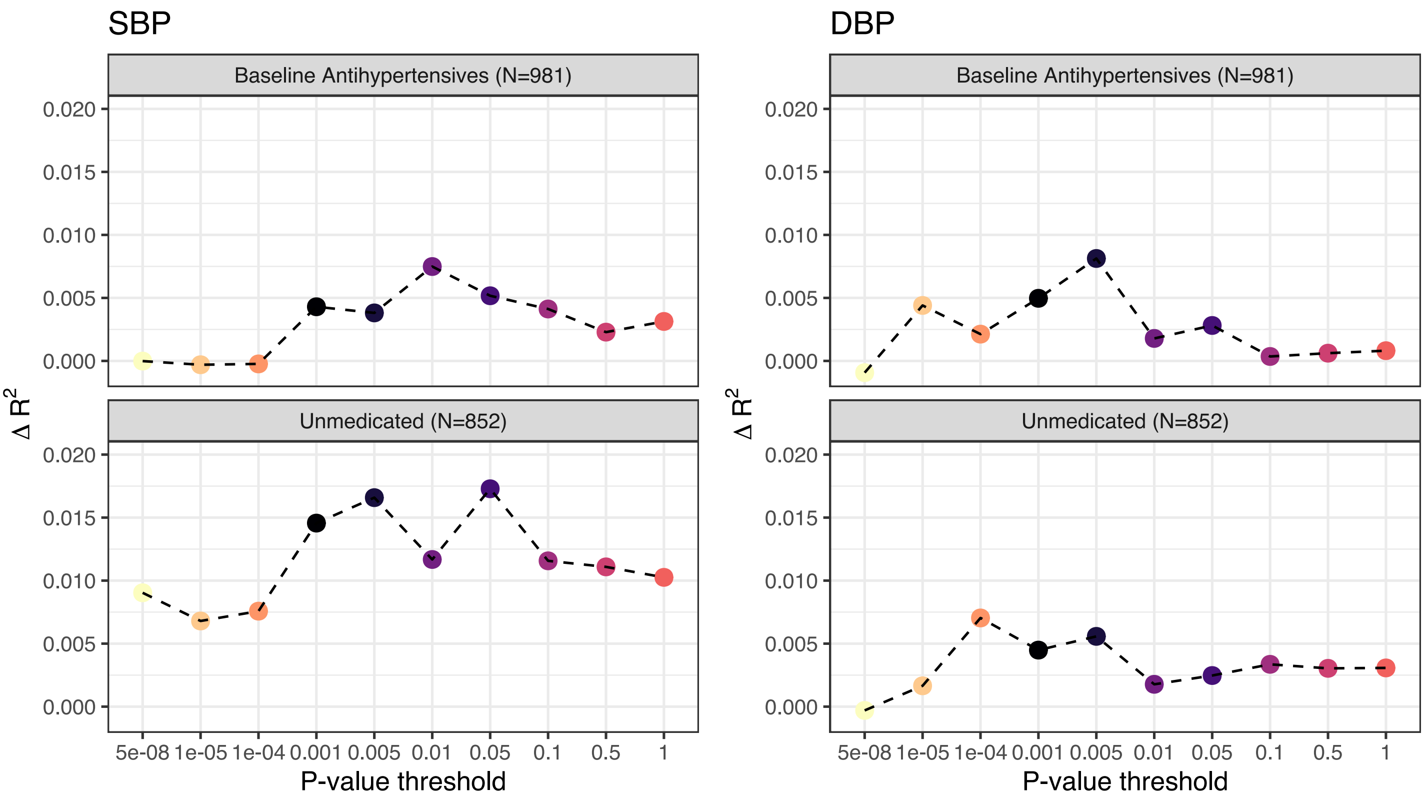

Figure S3. Tuning blood pressure polygenic scores (PGS) in the Hunter Community Study (HCS) cohort. Tuning genome-wide polygenic scores for SBP and DBP amongst medicated (antihypertensives) and unmedicated participants with measured blood pressure in the HCS. The variance explained between the full and covariate only model (ΔR^2) for each *P* value threshold is plotted.

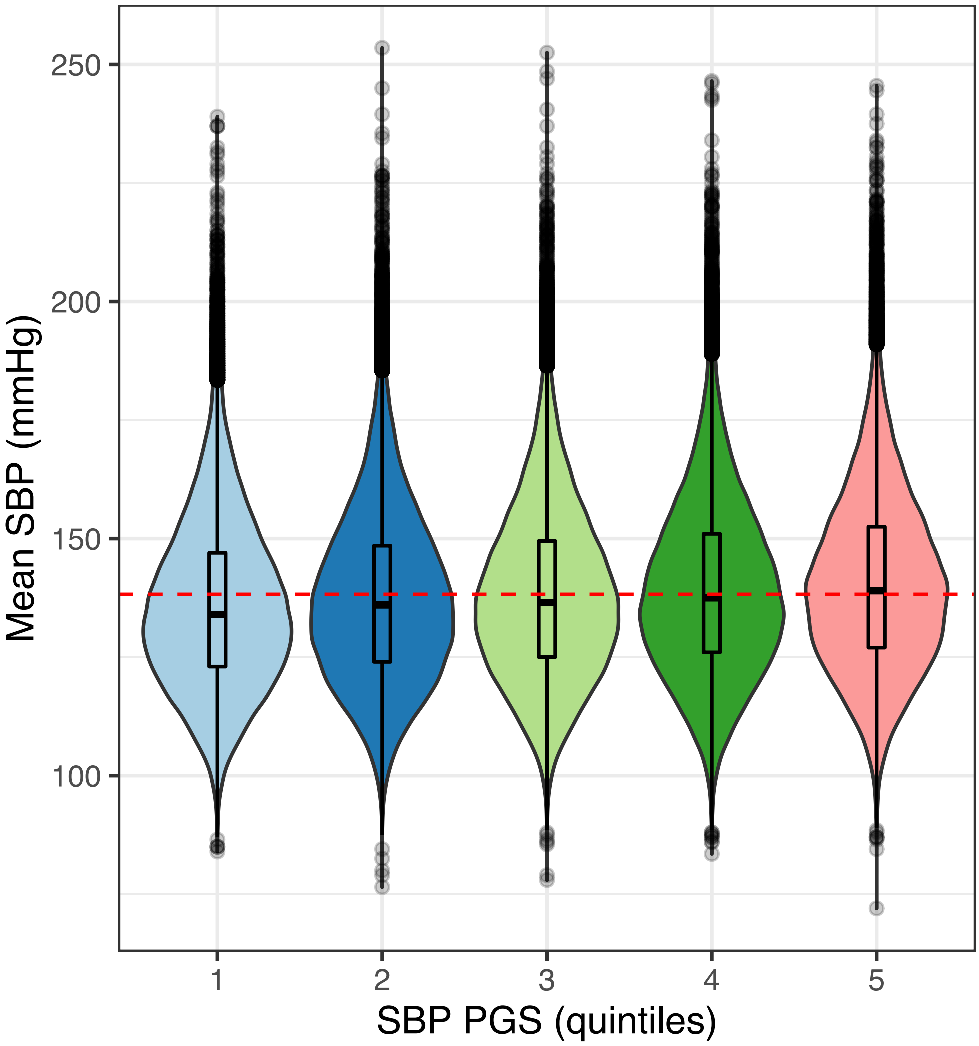

Figure S4. Association between a systolic blood pressure (SBP) polygenic score (PGS) and measured SBP. Violin plots, with overlaid box-and-whisker plots, of the distribution of measured SBP in each quintile of the SBP PGS in the UKBB cohort. The red dotted line denotes mean SBP in the entire cohort.

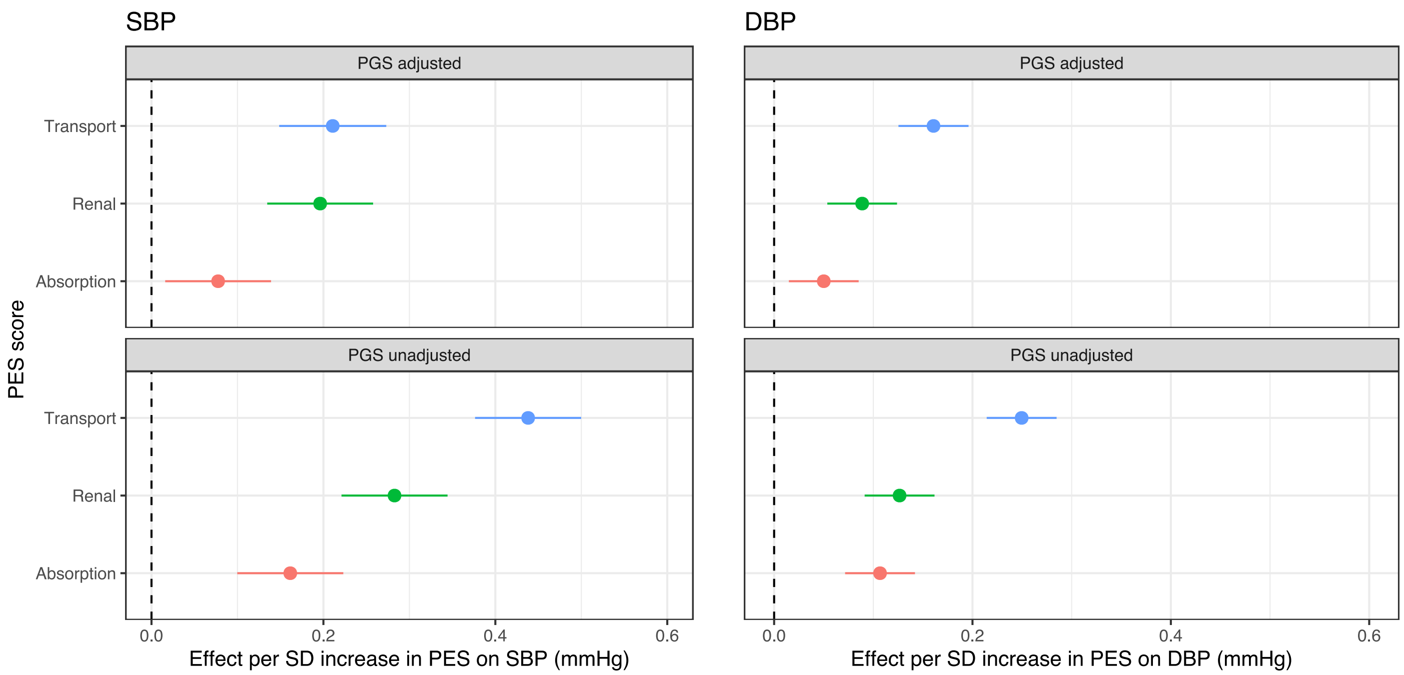

Figure S5. Effect sizes of sodium/potassium pharmagenic enrichment scores in the UKBB. Forest plot depicting the effect size (beta estimate with 95% confidence interval error bars) of the tuned PES in the UKBB on SBP and DBP, respectively. The top panel denotes models where genome wide PGS is covaried for, whilst the bottom panel are PGS unadjusted estimates.

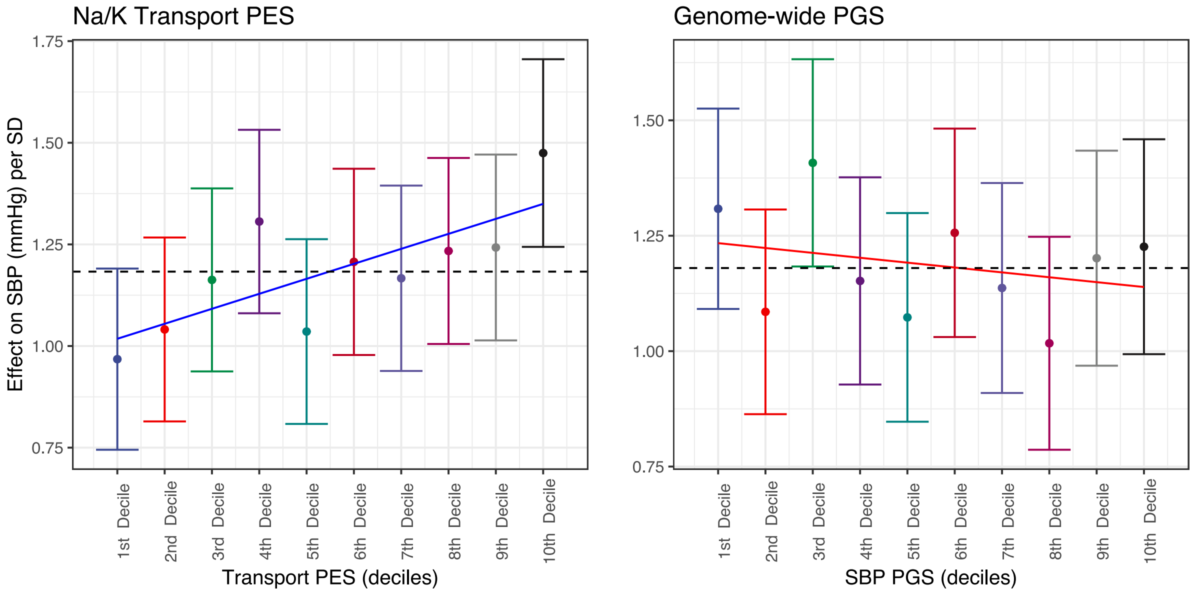

Figure S6. The estimated effect size of urinary sodium on SBP at differing values of sodium/potassium transport PES and genome-wide PGS. The estimated effect urinary sodium on SBP in the cohort upon splitting participants into deciles of the Na/K transport PES (left) or the genome wide PGS (right), with error bars denoting 95% confidence intervals of the estimate. These blood pressure effect sizes (in mmHg) are per standard deviation (standardised to be one in each decile) for urinary sodium. The dotted line denotes the mean urinary sodium/SBP effect size over all the deciles for either PES or PRS. A linear trend line is plotted between the per-decile beta estimates to visualise the trend of the effect sizes.

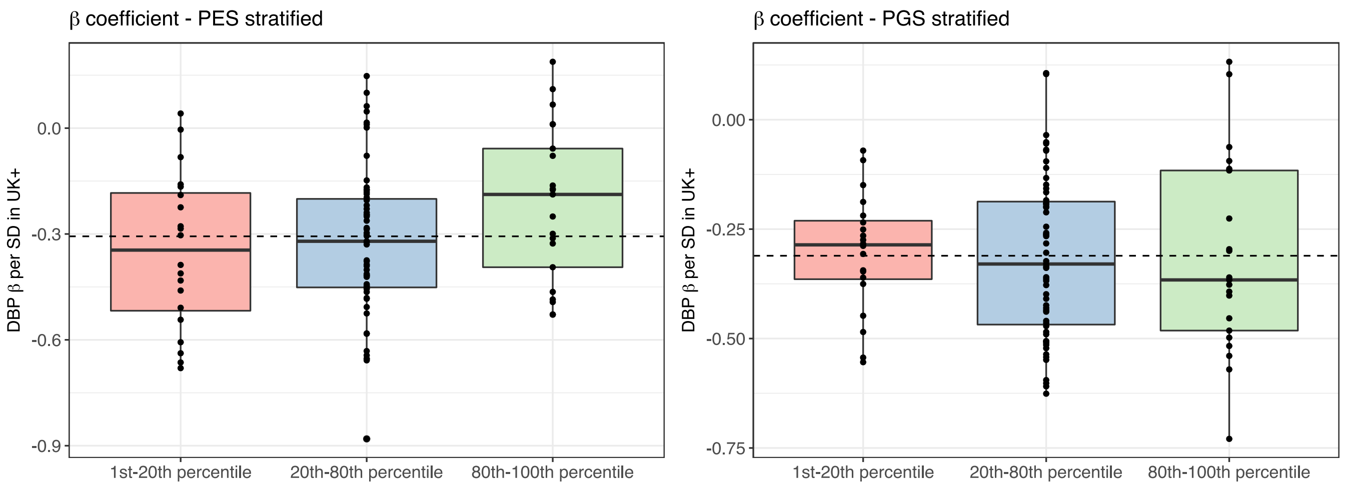

Figure S7. Exploring the association of urinary potassium with diastolic blood pressure with increasing sodium/potassium renal excretion *pharmagenic enrichment score*. On the left, the DBP effect size (mmHg) per standard deviation (SD) of scaled urinary potassium (SD = 1) in each percentile of the sodium/potassium renal excretion *pharmagenic enrichment score* (PES). The per-percentile estimates were grouped into low (1st-20th percentile), moderate (20th-80th percentile), and high (80th – top percentile). Box whisker plots were overlaid on the effect sizes in each group, denoting the median [+/- interquartile range) urinary sodium effect size for each group. The dotted line represents the mean SBP effect size across all percentiles. The righthand plot is analogous to the lefthand side but the percentiles instead represent percentiles of genome wide DBP polygenic score (PGS).

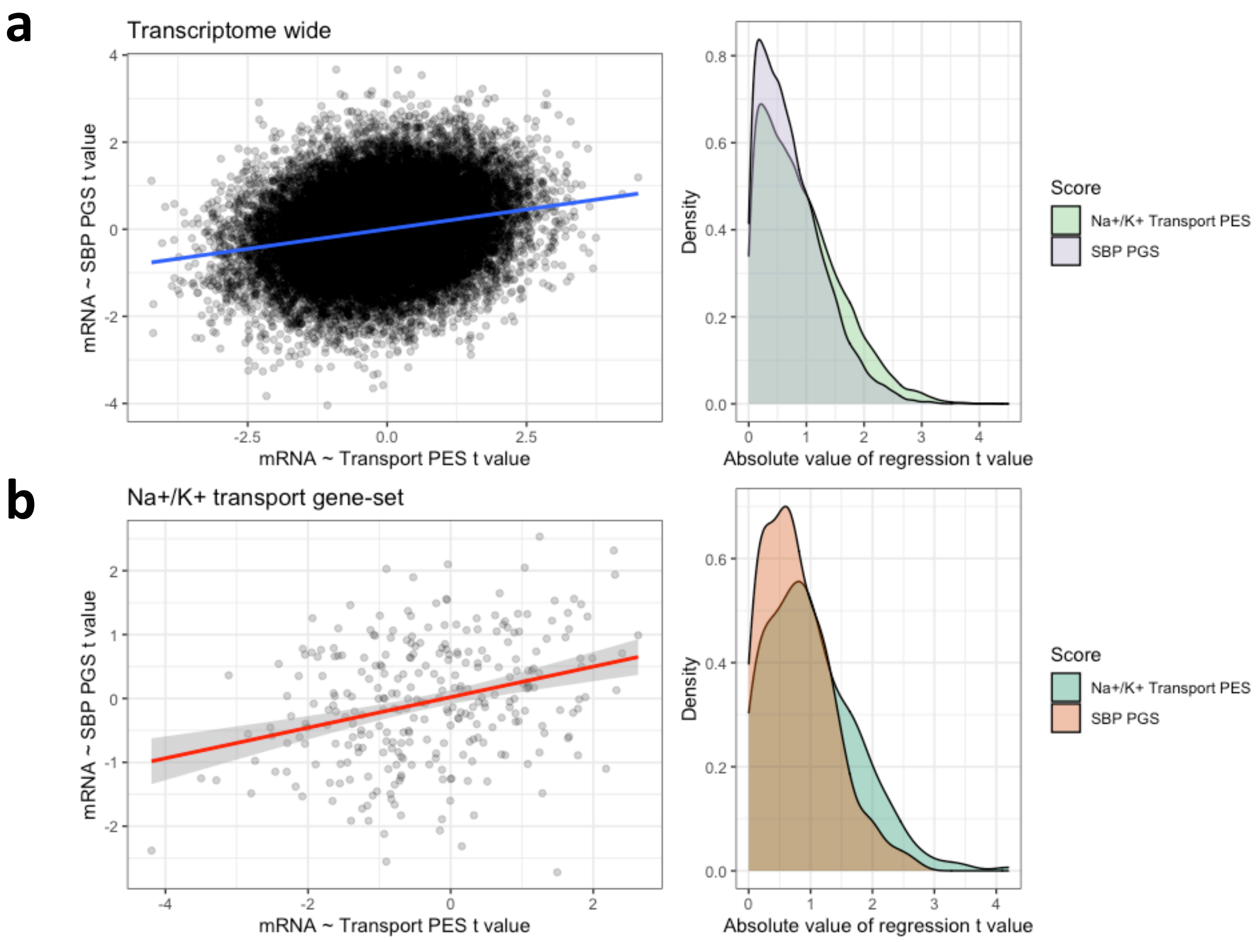

Figure S8. Transcriptional correlates of PES and PGS. The sodium/potassium transport PES and the genome wide PGS were regressed on the whole blood transcriptome. The correlation between the regression *t* value (beta/SE) for each gene is plotted transcriptome-wide (**a**) and specifically within the sodium/potassium transport gene-set (**b**). A kernel density estimation plot of the distribution of the absolute value of these regression *t* values is shown transcriptome-wide (**a**) and specifically within the sodium/potassium transport gene-set (**b**).

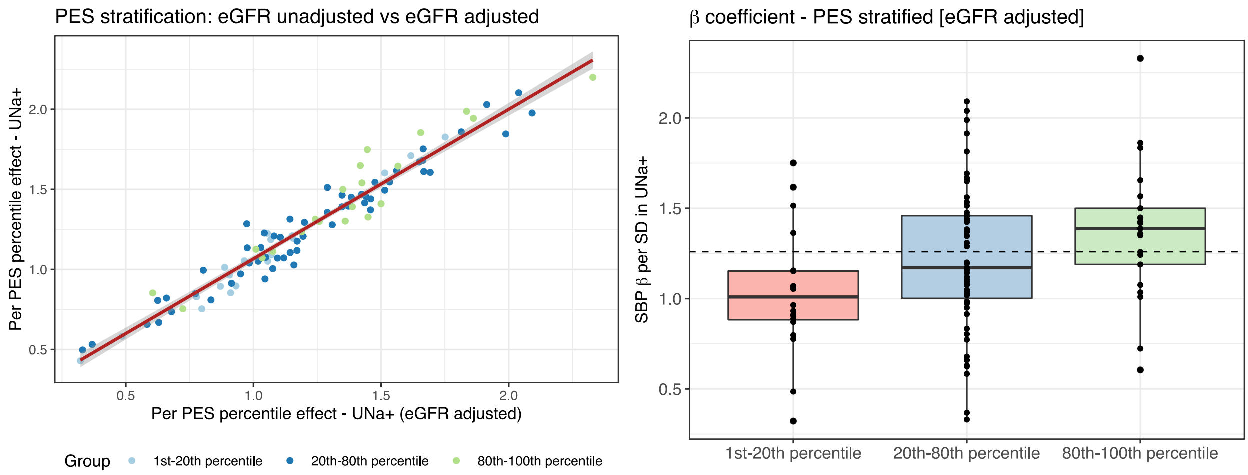

Figure S9. Consistency between PES percentile estimates of urinary sodium on systolic blood pressure upon covariation for estimated glomerular filtration rate (eGFR). The left hand-plot denotes the concordance between urinary sodium effect sizes with and without adjustment for eGFR. For visualisation purposes, the CKD-Epi eGFR is shown here but the concordance was almost identical (both r = 0.97) using the Stevens *et al.* equation derived eGFR. The right-hand plot represents the SBP effect size (mmHg) per standard deviation (SD) of scaled urinary sodium (SD = 1) in each percentile of the sodium/potassium transport pharmagenic enrichment score (PES), additionally adjusted for eGFR. The per-percentile estimates were grouped into low (1st-20th percentile), moderate (20th-80th percentile), and high (80th –top percentile). Box whisker plots were overlaid on the effect sizes in each group, denoting the median [+/- interquartile range) urinary sodium effect size for each group. The dotted line represents the mean SBP effect size across all percentiles.

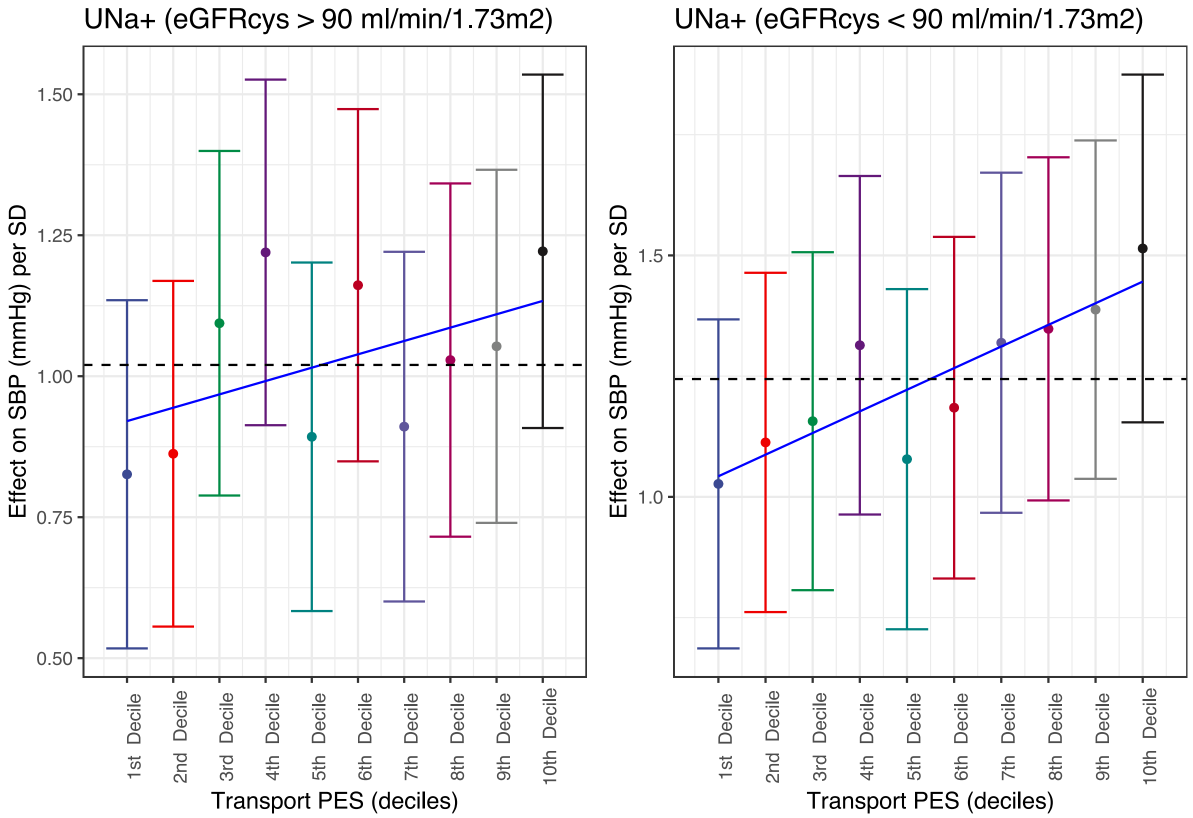

Figure S10. Estimated effect of urinary sodium per decile of the sodium/potassium transport PES – subsetted by eGFR (Stevens *et al.* eGFRCys). The estimated effect urinary sodium on SBP in the cohort upon splitting participants into deciles of the Na/K transport PES with error bars denoting 95% confidence intervals of the estimate. These blood pressure effect sizes (in mmHg) are per standard deviation (standardised to be one in each decile) for urinary sodium. The dotted line denotes the mean urinary sodium/SBP effect size over all the deciles for either PES or PRS. A linear trend line is plotted between the per-decile beta estimates to visualise the trend of the effect sizes. The left plot is indicative of these analyses performed in participants with an eGFR ≥ 90 ml/min/1.73m^2, whilst the righthand plot relates to participants with eGFR < 90 ml/min/1.73m^2.

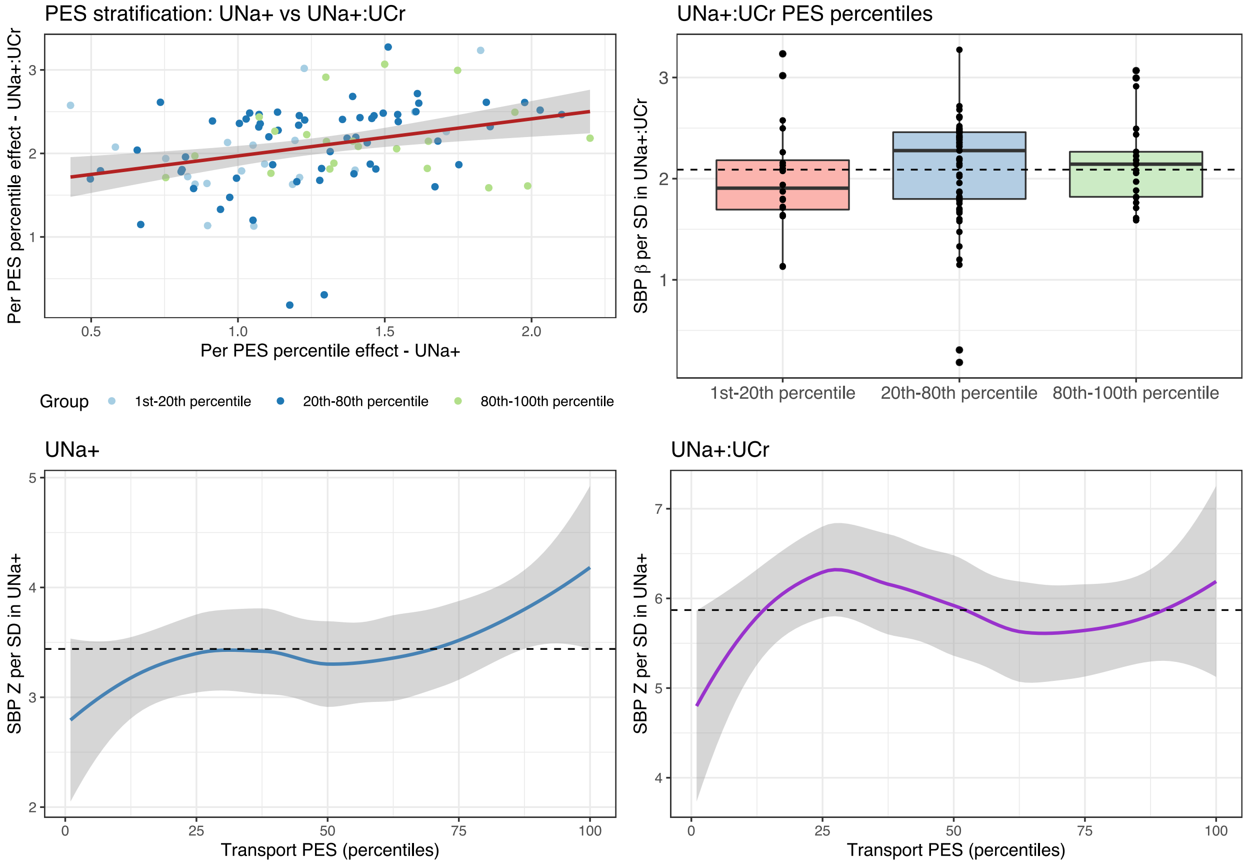

Figure S11. Consistency between PES percentile estimates of urinary sodium on systolic blood pressure versus urinary sodium-to-urinary creatinine ratio. The top left hand-plot denotes the concordance between urinary sodium (UNa^+) versus urinary sodium-to-creatinine ratio (UNa^+:UCr) effect sizes per PES percentile. The top right-hand plot represents the SBP effect size (mmHg) per standard deviation (SD) of scaled UNa^+:UCr (SD = 1) in each percentile of the sodium/potassium transport pharmagenic enrichment score (PES), additionally adjusted for eGFR. The per-percentile estimates were grouped into low (1st-20th percentile), moderate (20th-80th percentile), and high (80th –top percentile). Box whisker plots were overlaid on the effect sizes in each group, denoting the median [+/- interquartile range) urinary sodium effect size for each group. The dotted line represents the mean SBP effect size across all percentiles. The bottom plots represent a smoothed LOESS curve between the per-PES-percentile effect size of UNa^+ (left) and UNa^+:UCr (right).

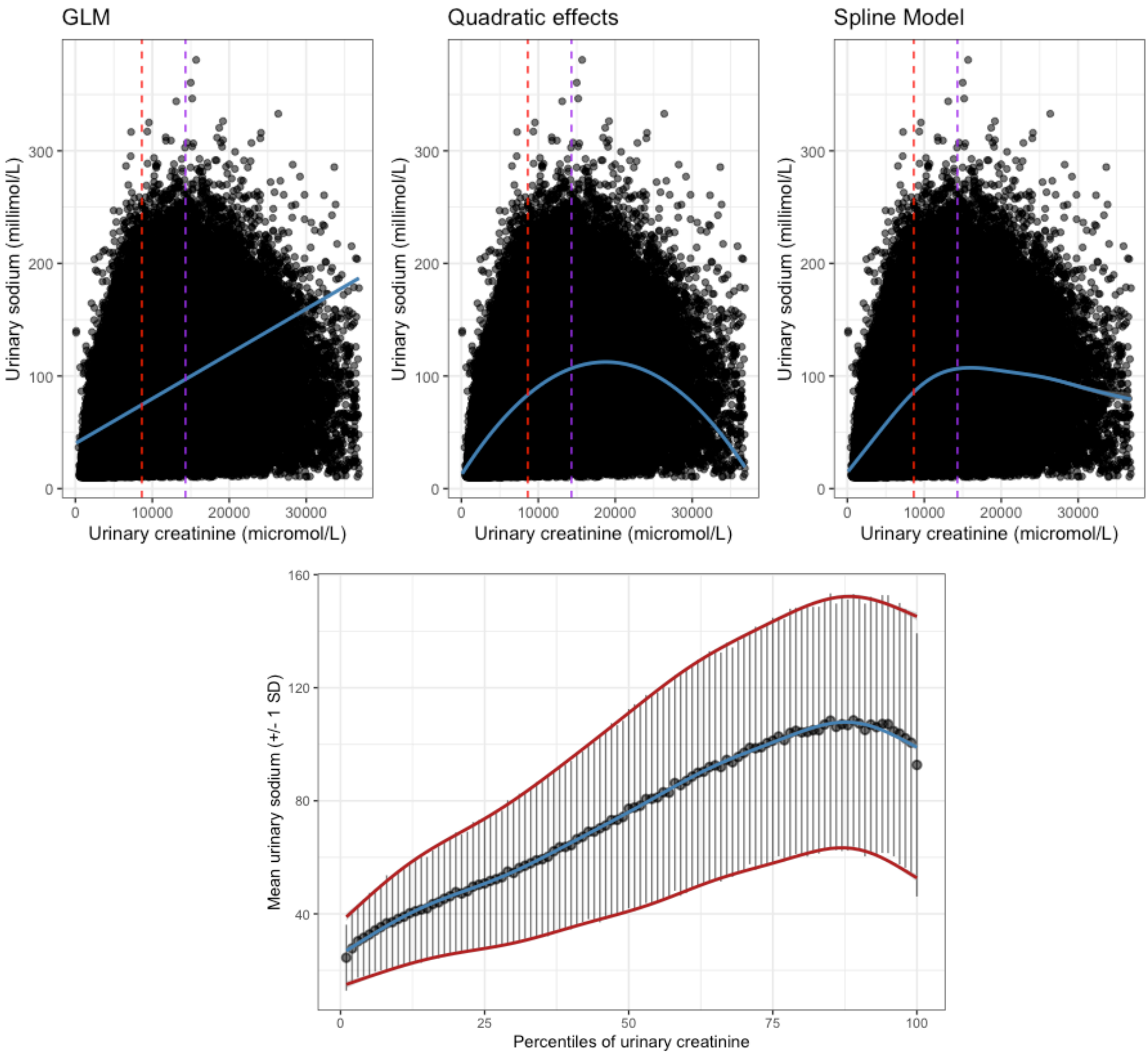

Figure S12. Exploring linear and non-linear features of the relationship between urinary creatinine and urinary sodium (unmedicated individuals). Creatinine outliers > 5 standard deviations above the mean winsorized for visualisation purposes. Red-dotted line denotes the mean urinary creatinine value, whilst the blue dotted line denotes values > 1 standard deviation above the mean. In the first model, we fit a conventional generalised linear model (glm) – with this smoothed curve shown in blue on the plot. Adjusted for age, age^2, sex, assessment centre, and assessment month the variance explained by a glm had Bayesian Information Criterion (BIC) of 2356578 . The second panel adds a quadratic which lowered the BIC, BIC = 2327628. Finally, we also fit a generalised additive model using thin plate regression splines (third panel) – the variance explained was similar to that of the glm with the quadratic term but with a lower

BIC, BIC = 2325672. In general, we see evidence of a linear relationship of urinary creatinine to urinary sodium up until values > 1 SD above the mean, with deviations from linearity thereafter. The bottom row panel visualises this relationship in a simplified form whereby urinary creatinine is split into percentiles and the mean (+/- 1 SD) is plotted (thin plate regression spline curve). This reinforces that the linearity of the urinary sodium ~ urinary correlation does start to dimmish at higher urinary creatinine values, although not with large magnitude through this method of analysis. An important consideration here is that this only summarises the association per-percentile and does oversimplify the relationship for ease of visualisation.

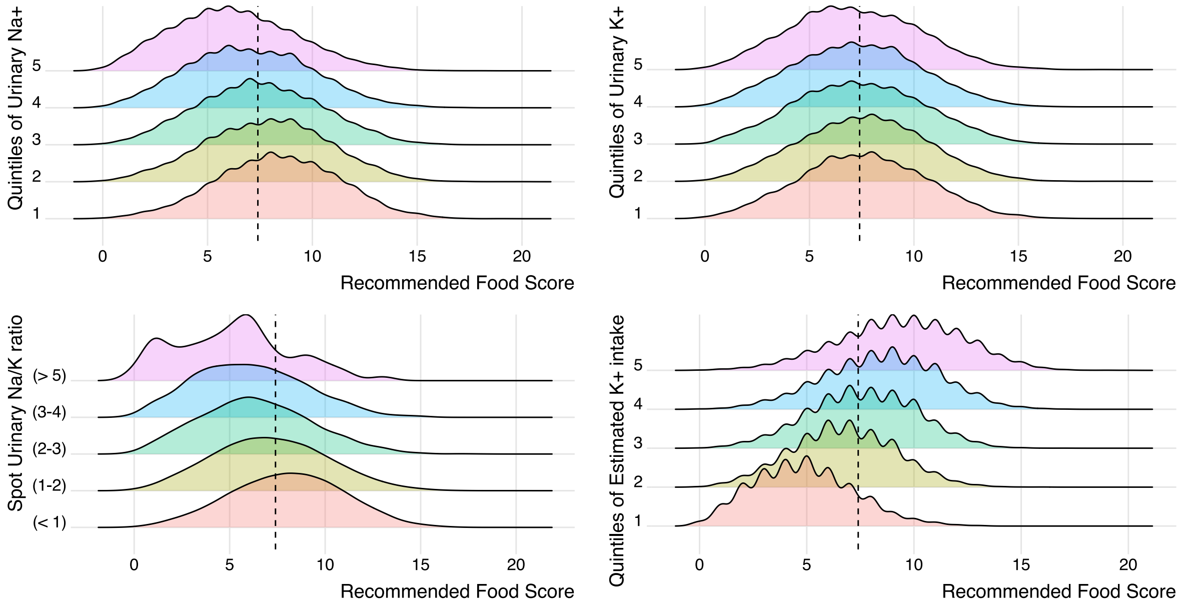

Figure S13. Ridgeline plot of the distribution (Kernel density estimation) of the recommended food score (RFS) amongst quantiles of outcome variables. The RFS distribution is plotted per quintile of urinary sodium (top left), urinary potassium (top right), and estimated potassium intake from the dietary questionnaire (bottom right). The bottom left is RFS distribution sodium/potassium ratio < 1, between 1 and 2, between 2-3, between 3-4, and greater than 5.
